# Supplementary material for: Model-based clustering for identifying disease-associated SNPs in case-control genome-wide association studies
Source: Sci Rep. 2019 Sep 23;9:13686. doi: 10.1038/s41598-019-50229-6 (PMC6757104; doi:10.1038/s41598-019-50229-6)
Supplement: Supplementary file 1 — Supplementary document [file 41598_2019_50229_MOESM1_ESM.pdf]

Supplementary Materials and Methods for "Model-based clustering for identifying disease-associated SNPs in case-control genome-wide association studies"

Yan Xu [1,†], Li Xing[2,†], Jessica Su[3], Xuekui Zhang[1,*,‡] and Weiliang Qiu[3,‡]

[1]: Department of Mathematics and Statistics, University of Victoria, 3800 Finnerty Road, Victoria, BC V8P 5C2 Canada

[2]: Department of Mathematics and Statistics, University of Saskatchewan, Saskatoon, SK, S7N 5E6 Canada

[3]: Channing Division of Network Medicine, Brigham and Women's Hospital/Harvard Medical School, 181 Longwood Avenue, Boston, MA 02115 USA

†: Joint First Authors contributed equally to this work

‡: Joint Senior Authors contributed equally to this work

∗: To whom correspondence should be addressed. Email: Xuekui@UVic.ca

Contents

Appendices **2**

A An overview of the proposed method **2**

B The mixture of 3-component Bayesian hierarchical models **3**

B.1 Bayesian hierarchical model for cluster 0 4

B.2 Bayesian hierarchical model for cluster + ... 5

B.3 Bayesian hierarchical model for cluster − 6

C The marginal density of a genotype profile **7**

C.1 The marginal density for cluster 0 7

C.2 The marginal density for cluster + ... 8

C.3 The marginal density for cluster − 9

D The EM algorithm to estimate model parameters **9**

D.1 Complete data likelihood function 10

D.2 E-step 11

D.3 M-step 12

E The choice of hyper-parameters α and β **13**

F Assigning SNP cluster membership **13**

G QC for real data analysis **13**

H Supplementary Figures **14**

I Supplementary Tables **17**

A An overview of the proposed method

Our method is to overcome the limitation of the traditional regression analysis when handling the high-dimensional problem (number of variables (SNPs) are much larger than the number of samples (subjects)). By regarding subjects as "variables" and SNPs as "samples", we will not have high-dimensional problem any more. This switch between variables and samples transforms the regression problem to SNP clustering problem. We used model-based clustering approach, instead of non-parametric clustering (such as hierarchical clustering and partitioning), to utilize biological information about the relationship of the minor allele frequency (MAF) between cases and controls. To cluster SNPs, we assume SNPs' genotypes follow a mixture of 3-component Bayesian hierarchical models. The 3 components correspond to 3 SNP clusters of interests in genome-wide association studies (GWASs): (1) SNPs positively associated with disease, (2) SNPs negatively associated with disease, and (3) SNPs not associated with disease.

This biological information greatly helps reduce the challenges in clustering SNPs so that we can set the number of clusters as 3 and know the relationship between MAF in cases and MAF in controls for the 3 clusters of SNPs.

For each SNP cluster, we assume the genotypes of a SNP follows a Bayesian hierarchical model to allow that different SNPs have different MAFs, while allowing to borrow information across SNPs. That is, although different SNPs have different MAFs within a cluster, these MAFs follow a common distribution. We can use all SNPs within a cluster to estimate the parameters of the common distribution.

We determine SNP cluster membership based on the posterior probability that a SNP belongs to a cluster given its genotype, which is a function of the marginal densities of the 3-component Bayesian hierarchical models. The posterior probabilities contain the parameters in the mixture model (mixture proportions and hyperparameters). We estimate the model

parameters via the EM algorithm, which is a commonly used parameter estimation method for mixture of distributions.

B The mixture of 3-component Bayesian hierarchical models

We assume there are G SNPs, n_x cases, and n_y controls. For a given SNP, we assume all cases have the same MAF and all controls also have the same MAF.

We are interested in three clusters of SNPs. Cluster 0 contains SNPs having the same MAF between cases and controls. Cluster + contains SNPs having higher MAF in cases and in controls. Cluster $-$ contains SNPs having lower MAF in cases and in controls.

Denote $S_{g,d,i}$ as the genotype of SNP g of patient i in group d, $g = 1, \ldots, G$, $i = 1, \ldots, n_d$, $d \in (x, y)$, x is the group of cases, and y is the group of controls. We assume all SNPs are bi-allelic and use additive coding for genotypes. $S_{g,d,i} = 0$ if the SNP g has zero minor allele (i.e., wild-type homozygous); $S_{g,d,i} = 1$ if the SNP g has one minor allele (i.e., heterozygous); $S_{g,d,i} = 2$ if the SNP g has two minor allele (i.e., mutant homozygous).

Denote $\theta_{g,d,k}$ as the MAF of SNP g in cluster $k \in \{0, +, -\}$ for subjects in group d. By assuming Hardy Weinberg Equilibrium, we have $Pr\left(S_{g,d,i} = 2\right) = \theta_{g,d,0}^2$, $Pr\left(S_{g,d,i} = 1\right) = 2\theta_{g,d,0}(1 - \theta_{g,d,0})$, and $Pr\left(S_{g,d,i} = 0\right) = (1 - \theta_{g,d,0})^2$.

In genome-wide association studies (GWASs), we are interested in detecting SNPs significantly associated with disease. We can further split the significant SNPs to two groups: positively disease-associated SNPs (i.e., the more minor allele is, the more chance to get the disease) and negatively disease-associated SNPs (i.e., the more minor allele is, the less chance to get the disease).

Denote π_k as the probability that the SNP g belongs to cluster k (i.e., $\pi_k = Pr\left(\text{SNP } g \in \text{ cluster } k\right)$), $k \in \{0, +, -\}$. We have $\pi_0 + \pi_+ + \pi_- = 1$.

Then the distribution of the genotype profile $\boldsymbol{S}_g = (S_{g,x,1}, \ldots, S_{g,x,n_x}, S_{g,y,1}, \ldots, S_{g,y,n_y})$

of the SNP g is the mixture of 3 distributions:

$$
\begin{aligned}
f\left(\boldsymbol{S}_g\right) =& Pr\left(\boldsymbol{S}_g \& \text{ SNP } g \text{ belongs to cluster 1}\right) \\
&+ Pr\left(\boldsymbol{S}_g \& \text{ SNP } g \text{ belongs to cluster 2}\right) \\
&+ Pr\left(\boldsymbol{S}_g \& \text{ SNP } g \text{ belongs to cluster 3}\right) \\
=& Pr\left(\boldsymbol{S}_g | \text{SNP } g \text{ belongs to cluster 1}\right) Pr\left(\text{SNP } g \text{ belongs to cluster 1}\right) \\
&+ Pr\left(\boldsymbol{S}_g | \text{SNP } g \text{ belongs to cluster 2}\right) Pr\left(\text{SNP } g \text{ belongs to cluster 2}\right) \\
&+ Pr\left(\boldsymbol{S}_g | \text{SNP } g \text{ belongs to cluster 3}\right) Pr\left(\text{SNP } g \text{ belongs to cluster 3}\right) \\
=& \pi_0 Pr\left(\boldsymbol{S}_g | \text{SNP } g \text{ belongs to cluster 1}\right) \\
&+ \pi_+ Pr\left(\boldsymbol{S}_g | \text{SNP } g \text{ belongs to cluster 2}\right) \\
&+ \pi_- Pr\left(\boldsymbol{S}_g | \text{SNP } g \text{ belongs to cluster 3}\right) \\
=& \pi_0 f_0\left(\boldsymbol{S}_g\right) + \pi_+ f_+\left(\boldsymbol{S}_g\right) + \pi_- f_-\left(\boldsymbol{S}_g\right),
\end{aligned}
\tag{B.1}
$$

where $f_k\left(\boldsymbol{S}_g\right) = Pr\left(\boldsymbol{S}_g | \text{SNP } g \text{ belongs to cluster k}\right)$.

Note that $f_k\left(\boldsymbol{S}_g\right)$ is a function of MAFs $\theta_{g,d,k}$. It is reasonable to assume the MAFs $\theta_{g,d,k}$ for SNPs within the same cluster follow the same distribution. Hence, we can use Bayesian hierarchical models to characterize the conditional distributions $\boldsymbol{S}_g$ given SNP g belongs to cluster k, $k = 0, +, -$.

B.1 Bayesian hierarchical model for cluster 0

For a SNP g in cluster 0, the MAF in cases is the same as that in controls. Denote $\theta_{g,0} = \theta_{g,x,i} = \theta_{g,y,j}$, $i = 1, \ldots, n_x$, $j = 1, \ldots, n_y$. We also use $S_{g,i}$ to denote the genotypes for subject i, $i = 1, \ldots, n_x + n_y$.

We assume the following Bayesian hierarchical model

$$
\begin{aligned}
S_{g,i} | \theta_{g,0} \sim & \, Multinomial\left(1, (\theta_{g,0}^2, 2\theta_{g,0}(1-\theta_{g,0}), (1-\theta_{g,0})^2)\right), \\
\theta_{g,0} \sim & \, Beta(\alpha, \beta), \\
i =& 1, \ldots, n_x + n_y.
\end{aligned}
\tag{B.2}
$$

The conditional density function is

$$
\begin{aligned}
f(S_{g,i} | \theta_{g,0}) = & (\theta_{g,0}^2)^{I(S_{g,i}=2)} \left[2\theta_{g,0}(1-\theta_{g,0})\right]^{I(S_{g,i}=1)} \left[(1-\theta_{g,0})^2\right]^{I(S_{g,i}=0)}, \\
i =& 1, \ldots, n_x + n_y.
\end{aligned}
\tag{B.3}
$$

The density function of the beta distribution $Beta(\alpha, \beta)$ is

$$h(\theta_{g,0}) = \frac{\theta_{g,0}^{\alpha-1}(1-\theta_{g,0})^{\beta-1}}{B(\alpha,\beta)}, \tag{B.4}$$

where B is the beta function.

B.2 Bayesian hierarchical model for cluster +

For a given SNP g in cluster +, we assume the following Bayesian hierarchical model:

$$\begin{aligned} S_{g,x,i}|\theta_{g,x,+} &\sim Multinomial\left(1, (\theta_{g,x,+}^2, 2\theta_{g,x,+}(1-\theta_{g,x,+}), (1-\theta_{g,x,+})^2)\right), \\ &\quad i = 1, \ldots, n_x, \\ S_{g,y,j}|\theta_{g,y,+} &\sim Multinomial\left(1, (\theta_{g,y,+}^2, 2\theta_{g,y,+}(1-\theta_{g,y,+}), (1-\theta_{g,y,+})^2)\right), \\ &\quad j = 1, \ldots, n_y, \\ (\theta_{g,x,+}, \theta_{g,y,+}) &\sim \text{a bivariate distribution } M_+. \end{aligned} \tag{B.5}$$

The conditional density functions are

$$\begin{aligned} f(S_{g,x,i}|\theta_{g,x,+}) &= (\theta_{g,x,+}^2)^{I(S_{g,x,i}=2)} \left[2\theta_{g,x,+}(1-\theta_{g,x,+})\right]^{I(S_{g,x,i}=1)} \left[(1-\theta_{g,x,+})^2\right]^{I(S_{g,x,i}=0)}, \\ &\quad i = 1, \ldots, n_x, \\ f(S_{g,y,j}|\theta_{g,y,+}) &= (\theta_{g,y,+}^2)^{I(S_{g,y,j}=2)} \left[2\theta_{g,y,+}(1-\theta_{g,y,+})\right]^{I(S_{g,y,j}=1)} \left[(1-\theta_{g,y,+})^2\right]^{I(S_{g,y,j}=0)}, \\ &\quad j = 1, \ldots, n_y. \end{aligned} \tag{B.6}$$

We assume the density function of the bivariate distribution M_+ has the following form:

$$f(\theta_{g,x,+}, \theta_{g,y,+}) = 2h(\theta_{g,x,+})h(\theta_{g,y,+})I(\theta_{g,x,+} > \theta_{g,y,+}), \tag{B.7}$$

which is "half-flat", that is

$$\begin{aligned} f(\theta_{g,x,+}, \theta_{g,y,+}) > 0, &\text{ when } \theta_{g,x,+} > \theta_{g,y,+}, \\ f(\theta_{g,x,+}, \theta_{g,y,+}) = 0, &\text{ when } \theta_{g,x,+} \leq \theta_{g,y,+}. \end{aligned} \tag{B.8}$$

B.3 Bayesian hierarchical model for cluster $-$

For a given SNP g in cluster $-$, we assume the following Bayesian hierarchical model:

$$\begin{aligned}
S_{g,x,i}|\theta_{g,x,-} &\sim Multinomial\left(1, (\theta_{g,x,-}^2, 2\theta_{g,x,-}(1-\theta_{g,x,-}), (1-\theta_{g,x,-})^2)\right), \\
i &= 1, \ldots, n_x, \\
S_{g,y,j}|\theta_{g,y,-} &= Multinomial\left(1, (\theta_{g,y,-}^2, 2\theta_{g,y,-}(1-\theta_{g,y,-}), (1-\theta_{g,y,-})^2)\right), \\
j &= 1, \ldots, n_y, \\
(\theta_{g,x,-}, \theta_{g,y,-}) &\sim \text{a bivariate distribution } M_-.
\end{aligned} \tag{B.9}$$

The conditional density functions are

$$\begin{aligned}
f(S_{g,x,i}|\theta_{g,x,-}) &= (\theta_{g,x,-}^2)^{I(S_{g,x,i}=2)} \left[2\theta_{g,x,-}(1-\theta_{g,x,-})\right]^{I(S_{g,x,i}=1)} \left[(1-\theta_{g,x,-})^2\right]^{I(S_{g,x,i}=0)}, \\
i &= 1, \ldots, n_x, \\
f(S_{g,y,j}|\theta_{g,y,-}) &= (\theta_{g,y,-}^2)^{I(S_{g,y,j}=2)} \left[2\theta_{g,y,-}(1-\theta_{g,y,-})\right]^{I(S_{g,y,j}=1)} \left[(1-\theta_{g,y,-})^2\right]^{I(S_{g,y,j}=0)}, \\
j &= 1, \ldots, n_y.
\end{aligned} \tag{B.10}$$

We assume the density function of the bivariate distribution M_- has the following form:

$$f(\theta_{g,x,-}, \theta_{g,y,-}) = 2h(\theta_{g,x,-})h(\theta_{g,y,-})I(\theta_{g,x,-} < \theta_{g,y,-}). \tag{B.11}$$

C The marginal density of a genotype profile

C.1 The marginal density for cluster 0

The marginal density of the genotype profile $\boldsymbol{S}_g$ can be written as follows.

$$
\begin{aligned}
\xi_0(\boldsymbol{S}_g) &= \int f\left(\boldsymbol{S}_g, \theta_{g,0}\right) d\theta_{g,0} \\
&= \int f\left(\boldsymbol{S}_g | \theta_{g,0}\right) h\left(\theta_{g,0}\right) d\theta_{g,0} \\
&= \int \left[\prod_{i=1}^{n_x+n_y} f\left(S_{g,i} | \theta_{g,0}\right)\right] h\left(\theta_{g,0}\right) d\theta_{g,0} \\
&= \int \left[\prod_{i=1}^{n_x+n_y} \left\{ (\theta_{g,0}^2)^{I(S_{g,i}=2)} \left[2\theta_{g,0}(1-\theta_{g,0})\right]^{I(S_{g,i}=1)} \left[(1-\theta_{g,0})^2\right]^{I(S_{g,i}=0)} \right\}\right] h\left(\theta_{g,0}\right) d\theta_{g,0} \\
&= \int \left\{ (\theta_{g,0}^2)^{n_{g,2}} \left[2\theta_{g,0}(1-\theta_{g,0})\right]^{n_{g,1}} \left[(1-\theta_{g,0})^2\right]^{n_{g,0}} \right\} \frac{\theta_{g,0}^{\alpha-1}(1-\theta_{g,0})^{\beta-1}}{\text{Beta}(\alpha,\beta)} d\theta_{g,0} \\
&= \frac{2^{n_{g,1}}}{\text{Beta}(\alpha,\beta)} \int \theta_{g,0}^{2n_{g,2}+n_{g,1}+\alpha-1}(1-\theta_{g,0})^{n_{g,1}+2n_{g,0}+\beta-1} d\theta_{g,0} \\
&= \frac{2^{n_{g,1}}\text{Beta}(\alpha_{g,0},\beta_{g,0})}{\text{Beta}(\alpha,\beta)}
\end{aligned}
\tag{C.1}
$$

where $\alpha_{g,0} = 2n_{g,2} + n_{g,1} + \alpha$, and $\beta_{g,0} = 2n_{g,0} + n_{g,1} + \beta$.

C.2 The marginal density for cluster +

The marginal density of the genotype profile $\boldsymbol{S}_g$ can be written as follows.

$$\begin{aligned}
\xi_+(\boldsymbol{S}_g) &= \iint f\left(\boldsymbol{S}_g, \theta_{g,x,+}, \theta_{g,y,+}\right) d\theta_{g,x,0} d\theta_{g,y,0} \\
&= \iint f\left(\boldsymbol{S}_g | \theta_{g,x,+}, \theta_{g,y,+}\right) f\left(\theta_{g,x,+}, \theta_{g,y,+}\right) d\theta_{g,x,0} d\theta_{g,y,0} \\
&= \iint f\left(\boldsymbol{S}_{g,x} | \theta_{g,x,+}\right) f\left(\boldsymbol{S}_{g,y} | \theta_{g,y,+}\right) f\left(\theta_{g,x,+}, \theta_{g,y,+}\right) d\theta_{g,x,0} d\theta_{g,y,0} \\
&= \iint f(\boldsymbol{S}_{g,x} | \theta_{g,x,+}) f(\boldsymbol{S}_{g,y} | \theta_{g,y,+}) 2h(\theta_{g,x,+}) h(\theta_{g,y,+}) I(\theta_{g,x,+} > \theta_{g,y,+}) d\theta_{g,x,+} d\theta_{g,y,+} \\
&= 2 \iint_{\theta_{g,x,+} > \theta_{g,y,+}} f(\boldsymbol{S}_{g,x} | \theta_{g,x,+}) h(\theta_{g,x,+}) f(\boldsymbol{S}_{g,y} | \theta_{g,y,+}) h(\theta_{g,y,+}) d\theta_{g,x,+} d\theta_{g,y,+} \\
&= 2 C_x C_y \iint_{\theta_{g,x,+} > \theta_{g,y,+}} \varphi(\theta_{g,x,+} | \boldsymbol{S}_{g,x}) \varphi(\theta_{g,y,+} | \boldsymbol{S}_{g,y}) d\theta_{g,x,+} d\theta_{g,y,+}, \\
&= 2 C_x C_y \Pr(V_x > V_y)
\end{aligned} \tag{C.2}$$

where

$$\begin{aligned}
C_x &= \int_{\theta_{g,x,+}} f(\boldsymbol{S}_{g,x} | \theta_{g,x,+}) h(\theta_{g,x,+}) d\theta_{g,x,+} = \frac{2^{n_{g,x,1}} \text{Beta}(\alpha_{g,x}, \beta_{g,x})}{\text{Beta}(\alpha, \beta)}, \\
C_y &= \int_{\theta_{g,y,+}} f(\boldsymbol{S}_{g,y} | \theta_{g,y,+}) h(\theta_{g,y,+}) d\theta_{g,y,+} = \frac{2^{n_{g,y,1}} \text{Beta}(\alpha_{g,y}, \beta_{g,y})}{\text{Beta}(\alpha, \beta)}, \\
\varphi(\theta_{g,x,+} | \boldsymbol{S}_{g,x}) &= \text{Beta}(\alpha_{g,x}, \beta_{g,x}), \\
\varphi(\theta_{g,y,+} | \boldsymbol{S}_{g,y}) &= \text{Beta}(\alpha_{g,y}, \beta_{g,y}),
\end{aligned} \tag{C.3}$$

with $\alpha_{g,x} = 2n_{g,x,2} + n_{g,x,1} + \alpha, \beta_{g,x} = 2n_{g,x,0} + n_{g,x,1} + \beta, \alpha_{g,y} = 2n_{g,y,2} + n_{g,y,1} + \alpha, \beta_{g,y} = 2n_{g,y,0} + n_{g,y,1} + \beta$.

Here V_x and V_y are independent variables distributed according to $\varphi(\theta_{g,x,+} | \boldsymbol{S}_{g,x})$ and $\varphi(\theta_{g,y,+} | \boldsymbol{S}_{g,y})$ respectively.

Thus $Pr(V_x > V_y)$ can be calculated as follows:

$$\begin{aligned} Pr(V_x - V_y > 0) &= \int_0^1 \int_{v_y}^1 \text{Beta}(\alpha_{g,x}, \beta_{g,x})\text{Beta}(\alpha_{g,y}, \beta_{g,y}) dv_x dv_y \\ &= \int_0^1 \left[\int_{v_y}^1 \text{Beta}(\alpha_{g,x}, \beta_{g,x}) dv_x \right] \text{Beta}(\alpha_{g,y}, \beta_{g,y}) dv_y \\ &= 1 - \int_0^1 F_{V_x}(v_y)\text{Beta}(\alpha_{g,y}, \beta_{g,y}) dv_y, \end{aligned} \tag{C.4}$$

where $F_{V_x}(\cdot)$ is Beta cumulative distribution function (CDF). $Pr(V_x < V_y)$ is just $1 - Pr(V_x > V_y)$. Eq. C.4 can be calculated by basic inbuilt functions (e.g., 'pbeta','dbeta', and 'integrate') in R.

C.3 The marginal density for cluster $-$

Similarly, we can calculate the marginal density of the genotype profile $\boldsymbol{S}_g$ for a given SNP g in cluster $-$, which is equal to

$$\xi_-(\boldsymbol{S}_g) = 2C_x C_y \int_0^1 F_{V_x}(v_y)\text{Beta}(\alpha_{g,y}, \beta_{g,y}) dv_y. \tag{C.5}$$

D The EM algorithm to estimate model parameters

We used an EM algorithm to estimate the model parameters $(\pi_0, \pi_+, \pi_-, \alpha, \beta)$. The first step of the EM algorithm is to introduce "missing values" $z_{g,k}$, $g = 1, \ldots, G$, $k = 0, +, -$, which are binary variables indicating if SNP g belongs to cluster k. That is, $z_{g,k} = 1$ if SNP g is in cluster k; $z_{g,k} = 0$ if SNP g is not in cluster k.

We assume that $z_{g,k}$ are Bernoulli distributed with parameter π_k, $k = 0, +, -$. That is, $\pi_k = Pr\left(z_{g,k} = 1\right)$.

To stabilize the estimate of π_k when π_k is very small, we assume that the mixture proportions $(\pi_0, \pi_+, \pi_-)^T$ are Dirichlet distributed with parameters $\boldsymbol{b} = (b_0, b_+, b_-)^T$, with density function

$$Dir(\boldsymbol{\pi}|\boldsymbol{b}) = \frac{\Gamma\left(\sum_{k\in\{0,+,-\}} b_k\right)}{\prod_{k\in\{0,+,-\}} \Gamma(b_k)} \prod_{k\in\{0,+,-\}} \pi_k^{b_k - 1}.$$

D.1 Complete data likelihood function

The complete data are $\{\boldsymbol{S}, \boldsymbol{z}, \boldsymbol{\pi}\}$, where $\boldsymbol{S} = \{\boldsymbol{S}_1, \ldots, \boldsymbol{S}_G\}$, $\boldsymbol{z} = \{z_{1,0}, \ldots, z_{G,0}, z_{1,+}, \ldots, z_{G,+}, z_{1,-}, \ldots, z_{G,-}\}$, and $\boldsymbol{\pi} = \{\pi_0, \pi_+, \pi_-\}$.

The complete data likelihood function is

$$
\begin{aligned}
& f(\boldsymbol{S}, \boldsymbol{z}, \boldsymbol{\pi}) \\
=& f(\boldsymbol{S}, \boldsymbol{z}|\boldsymbol{\pi}) f(\boldsymbol{\pi}) \\
=& \left[\prod_{g=1}^{G} f(\boldsymbol{S}_g, \boldsymbol{z}_g|\boldsymbol{\pi})\right] f(\boldsymbol{\pi}) \\
=& \left\{\prod_{g=1}^{G} \prod_{k\in\{0,+,-\}} [f(\boldsymbol{S}_g, \boldsymbol{z}_g|\boldsymbol{\pi})]^{z_{g,k}}\right\} f(\boldsymbol{\pi}) \\
=& \left\{\prod_{g=1}^{G} \prod_{k\in\{0,+,-\}} \pi_k^{z_{g,k}} [f(\boldsymbol{S}_g|\boldsymbol{z}_g = z_{g,k}, \boldsymbol{\pi})]^{z_{g,k}}\right\} \frac{\Gamma(b_0 + b_+ + b_-)}{\Gamma(b_0)\Gamma(b_+)\Gamma(b_-)} \pi_0^{(b_0-1)} \pi_+^{(b_+-1)} \pi_-^{(b_--1)} \\
=& \frac{\Gamma(b_0 + b_+ + b_-)}{\Gamma(b_0)\Gamma(b_+)\Gamma(b_-)} \pi_0^{\left[(b_0-1)+\sum_{g=1}^{G} z_{g,0}\right]} \pi_+^{\left[(b_+-1)+\sum_{g=1}^{G} z_{g,+}\right]} \pi_-^{\left[(b_--1)+\sum_{g=1}^{G} z_{g,-}\right]} \\
& \cdot \left\{\prod_{g=1}^{G} \prod_{k\in\{0,+,-\}} [f(\boldsymbol{S}_g|\boldsymbol{z}_g = z_{g,k}, \boldsymbol{\pi})]^{z_{g,k}}\right\}.
\end{aligned}
\tag{D.1}
$$

The complete data log-likelihood is

$$\begin{aligned}
\ell\left(\boldsymbol{S},\boldsymbol{Z}|\boldsymbol{\pi},\alpha,\beta,\boldsymbol{b}\right) &= \sum_{g=1}^{G}\sum_{k\in\{0,+,-\}}\{z_{g,k}\log\xi_k(\boldsymbol{S}_g|\alpha,\beta)+z_{g,k}\log\pi_k\} \\
&+\log\left(\frac{\Gamma\left(\sum_{k\in\{0,+,-\}}b_k\right)}{\prod_{k\in\{0,+,-\}}\Gamma(b_k)}\right)+\sum_{k\in\{0,+,-\}}(b_k-1)\log(\pi_k) \\
&= \sum_{g=1}^{G}\{z_{g,0}\log\xi_0(\boldsymbol{S}_g|\alpha,\beta)+z_{g,+}\log\xi_+(\boldsymbol{S}_g|\alpha,\beta)+z_{g,-}\log\xi_-(\boldsymbol{S}_g|\alpha,\beta)\} \\
&+\sum_{g=1}^{G}\{z_{g,0}\log\pi_0+z_{g,+}\log\pi_++z_{g,-}\log\pi_-\} \\
&+\log\left(\frac{\Gamma\left(\sum_{k\in\{0,+,-\}}b_k\right)}{\prod_{k\in\{0,+,-\}}\Gamma(b_k)}\right)+\sum_{k\in\{0,+,-\}}(b_k-1)\log(\pi_k).
\end{aligned} \tag{D.2}$$

D.2 E-step

Note that $z_{g,k}$, $g=1,\ldots,G$, $k=0,+,-$, are unknown, hence are "missing values". In the E step, we integrate out $z_{g,k}$ from the complete data log likelihood function. That is, we calculate the expected complete data log-likelihood conditional on observations $\boldsymbol{S}$ and current estimates of the parameters $\hat{\boldsymbol{\pi}}$.

$$\begin{aligned}
Q(\boldsymbol{\pi},\alpha,\beta;\hat{\boldsymbol{\pi}},\alpha,\beta) &= E_z[\ell(\boldsymbol{S},\boldsymbol{Z}|\boldsymbol{\pi},\alpha,\beta,\boldsymbol{b})|\boldsymbol{S},\hat{\boldsymbol{\pi}},\alpha,\beta,\boldsymbol{b}] \\
&= \sum_{g=1}^{G}\sum_{k\in\{0,+,-\}}\gamma(z_{g,k}|\boldsymbol{S}_g,\hat{\boldsymbol{\pi}},\alpha,\beta)\{\log\pi_k+\log\xi_k(\boldsymbol{S}_g|\alpha,\beta)\} \\
&+\log\left(\frac{\Gamma(\sum_{k\in\{0,+,-\}}b_k)}{\prod_{k\in\{0,+,-\}}\Gamma(b_k)}\right)+\sum_{k\in\{0,+,-\}}(b_k-1)\log(\pi_k)
\end{aligned} \tag{D.3}$$

where $\gamma(z_{g,k})$ is the posterior probability (responsibility) given observed data ($\boldsymbol{S}_g$) and estimates of the parameters ($\hat{\boldsymbol{\pi}}$):

$$\begin{aligned}
\gamma(z_{g,k}) =& \gamma(z_{g,k}|\boldsymbol{S}_g, \hat{\boldsymbol{\pi}}, \alpha, \beta) \\
=& E\left[z_{g,k}|\boldsymbol{S}_g, \hat{\boldsymbol{\pi}}, \alpha, \beta\right] \\
=& Pr\left[z_{g,k} = 1|\boldsymbol{S}_g, \hat{\boldsymbol{\pi}}, \alpha, \beta\right] \\
=& \frac{Pr\left[z_{g,k} = 1, \boldsymbol{S}_g, \hat{\boldsymbol{\pi}}|\alpha, \beta\right]}{Pr\left[\boldsymbol{S}_g, \hat{\boldsymbol{\pi}}|\alpha, \beta\right]} \\
=& \frac{Pr\left[\boldsymbol{S}_g|z_{g,k} = 1, \hat{\boldsymbol{\pi}}, \alpha, \beta\right] Pr\left[z_{g,k} = 1|\hat{\boldsymbol{\pi}}, \alpha, \beta\right] Pr\left[\hat{\boldsymbol{\pi}}|\alpha, \beta\right]}{\sum_{j\in\{0,+,-\}} Pr\left[\boldsymbol{S}_g|z_{g,j} = 1, \hat{\boldsymbol{\pi}}, \alpha, \beta\right] Pr\left[z_{g,j} = 1|\hat{\boldsymbol{\pi}}, \alpha, \beta\right] Pr\left[\hat{\boldsymbol{\pi}}|\alpha, \beta\right]} \\
=& \frac{\hat{\pi}_k \xi_k(\boldsymbol{S}_g|\alpha, \beta)}{\sum_{j\in\{0,+,-\}} \hat{\pi}_j \xi_j(\boldsymbol{S}_g|\alpha, \beta)}.
\end{aligned} \tag{D.4}$$

D.3 M-step

In the M step, we maximize the expectation of complete data log-likelihood over $\boldsymbol{\pi}$. Recall $\pi_0 + \pi_+ + \pi_- = 1$ and $\sum_{g=1}^{G}\{\gamma(z_{g,0}) + \gamma(z_{g,+}) + \gamma(z_{g,-})\} = 1$. We take partial derivative of Q w.r.t π_0, π_+, π_-:

$$\begin{aligned}
\frac{\partial Q}{\partial \pi_0} &= \sum_{g=1}^{G}\left(\frac{\gamma(z_{g,0})}{\pi_0} - \frac{\gamma(z_{g,-})}{1-\pi_0-\pi_+}\right) + \frac{b_0 - 1}{\pi_0} - \frac{b_- - 1}{1-\pi_0-\pi_+} \\
\frac{\partial Q}{\partial \pi_+} &= \sum_{g=1}^{G}\left(\frac{\gamma(z_{g,+})}{\pi_+} - \frac{\gamma(z_{g,-})}{1-\pi_0-\pi_+}\right) + \frac{b_+ - 1}{\pi_+} - \frac{b_- - 1}{1-\pi_0-\pi_+}.
\end{aligned} \tag{D.5}$$

By solving the equations above and $\pi_+ + \pi_- + \pi_0 = 1$, we obtain:

$$\begin{aligned}
\pi_0^{\text{new}} &= \frac{\sum_{g=1}^{G}\gamma(z_{g,0}) + b_0 - 1}{G + b_0 + b_+ + b_- - 3} \\
\pi_+^{\text{new}} &= \frac{\sum_{g=1}^{G}\gamma(z_{g,+}) + b_+ - 1}{G + b_0 + b_+ + b_- - 3} \\
\pi_-^{\text{new}} &= \frac{\sum_{g=1}^{G}\gamma(z_{g,-}) + b_- - 1}{G + b_0 + b_+ + b_- - 3}
\end{aligned} \tag{D.6}$$

α and β are pre-specified hyper-parameters, and their choices are mentioned in the next session. We repeat updating $\boldsymbol{\pi}$ and $\boldsymbol{Z}$ and stop till convergence which is defined as the squared difference between the proposed $\boldsymbol{\pi}$ and current $\boldsymbol{\pi}$ is smaller than $1/G^2$. Note we do

not have to calculate $\frac{\partial Q}{\partial \pi_-}$, since π_- can be calculated as $1 - \pi_+ - \pi_0$.

E The choice of hyper-parameters α and β

We used moment matching methods to determine the values of the hyper-parameters α and β. The idea of moment matching is to use the prior, $\text{Beta}(\alpha, \beta)$, to approximate either the empirical distribution of MAF from the data or the truncated $\text{Beta}(2, 5)$. This approximation is done based on matching the mean, μ, and the variance, σ of the target distributions with the mean and variance of $\text{Beta}(\alpha, \beta)$, particularly by solving the equations below.

$$\begin{aligned} \alpha =& \mu \left(\frac{\mu(1-\mu)}{\sigma} - 1 \right) \\ \beta =& (1-\mu) \left(\frac{\mu(1-\mu)}{\sigma} - 1 \right). \end{aligned} \tag{E.1}$$

If the target distribution is the empirical distribution of MAF, we use the sample mean of the MAF $\overline{\theta} = \frac{1}{G} \sum_{g=1}^{G} \hat{\theta}_g$ with $\hat{\theta}_g = \frac{2n_{g,2} + n_{g,1}}{2(n_x + n_y)}$, and its sample variance $\frac{1}{G-1} \sum_{g=1}^{G} (\hat{\theta}_g - \overline{\theta})^2$ for moment matching. If the target distribution is the truncated $\text{Beta}(2, 5)$, its mean and variance are calculated based on Nadarajah and Kotz (2006).

F Assigning SNP cluster membership

To assign a SNP to one of the three clusters (0, +, and −), we first calculate $\gamma(z_{g,k})$, the posterior probability (responsibility) given observed data ($\boldsymbol{S}_g$) and estimates of the parameters ($\hat{\boldsymbol{\pi}}$) (see Formula D.4).

A straightforward decision rule about cluster membership is to assign each SNP to the cluster with the highest posterior probability, i.e., assign SNP g to the cluster corresponding to the largest value of $\gamma(z_{g,0})$, $\gamma(z_{g,+})$, and $\gamma(z_{g,-})$.

Alternatively, we can applied Yuan and Kendziorski's (2006) method Yuan and Kendziorski (2006) that is based on estimated FDR.

G QC for real data analysis

We performed the following 5 steps to control the quality of the SNP data.

Step 1: We excluded 62,301 SNPs, which are unannotated SNPs, mitochondrial SNPs, SNPs on sex chromosomes, or SNPs with zero variation.

Step 2: We excluded 16,396 SNPs with large proportions ($> 5\%$) of missing values.

Step 3: We excluded 490,750 SNPs with low genotype frequency ($< 5\%$) in either the case group or the control group. In Magrangeas et al. (2016), they excluded 483,984 SNPs having low genotype frequency ($< 5\%$) in the total sample.

Step 4: We excluded 657 SNPs, which did not pass the Hardy-Weinberg equilibrium (HWE) test based on p value $< 10^{-5}$.

Step 5: We keep the SNPs that passed QC both in the discovery set (GSE65777) and in the validation set (GSE66903). Therefore, 92,146 SNPs were excluded due to this criteria. Note that Magrangeas et al. (2016) keep the SNPs that only pass QC on discovery set, hence SNPs cannot pass QC on validation set is still included in analysis. This change helps us to screen out a lot more SNPs that cannot be validated even they were discovered.

H Supplementary Figures

Figure S1-S5 present simulation results for different SNP sizes with different numbers of effective SNPs using 4 different MAF distributions for data generation and 2 different sample sizes respectively. The upper and lower 4 rows contain results of simulated data with 200 samples and 1,000 samples respectively. The left panel shows truncated MAF distributions for data generation (solid line) and prior distributions for analysis (approximated beta distributions via moment matching: dashed lines are Beta-approximations of truncated Beta(2,5) and dotted lines are Beta- approximations of empirical distributions estimated from data). The middle panel shows boxplots for FDR and the vertical dashed line represents the nominal level (0.05). The right panel shows boxplots for paired difference of sensitivity between our method (using truncated Beta(2,5) or empirical distribution for analysis) and the SNP-wise approach, and the vertical dashed line represents 0 (i.e., same performance between our method and the SNP-wise approach). White boxes represent the SNP-wise approach, light grey boxes represent our method using truncated Beta(2,5) for analysis, and dark grey boxes represent our method using empirical distributions for analysis.

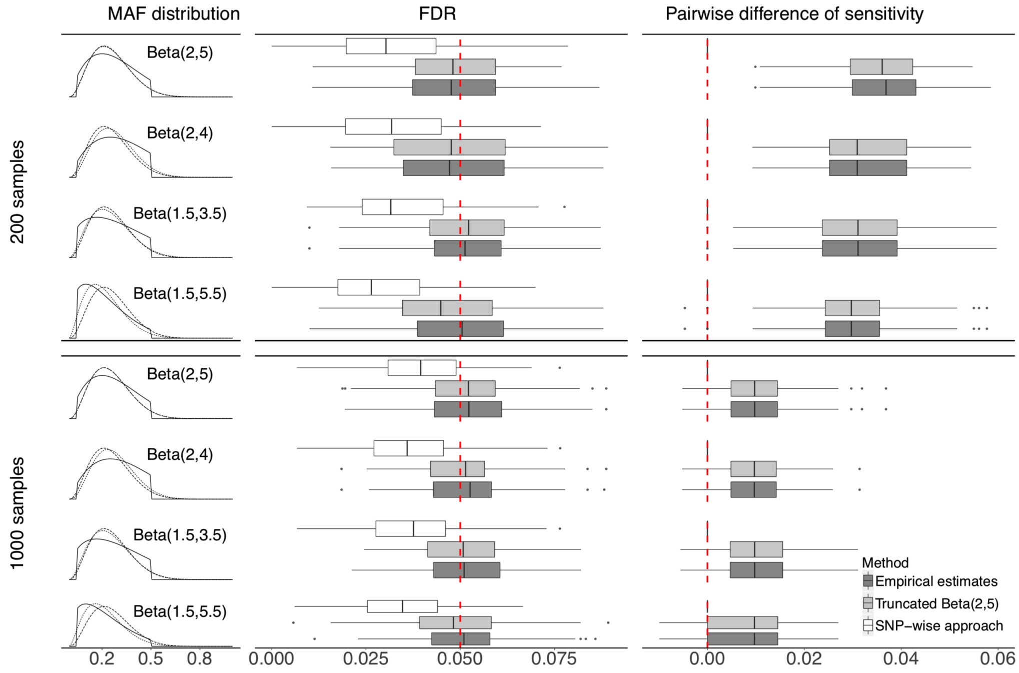

Figure S1: Simulation results for 1000 total SNPs with 200 effective SNPs.

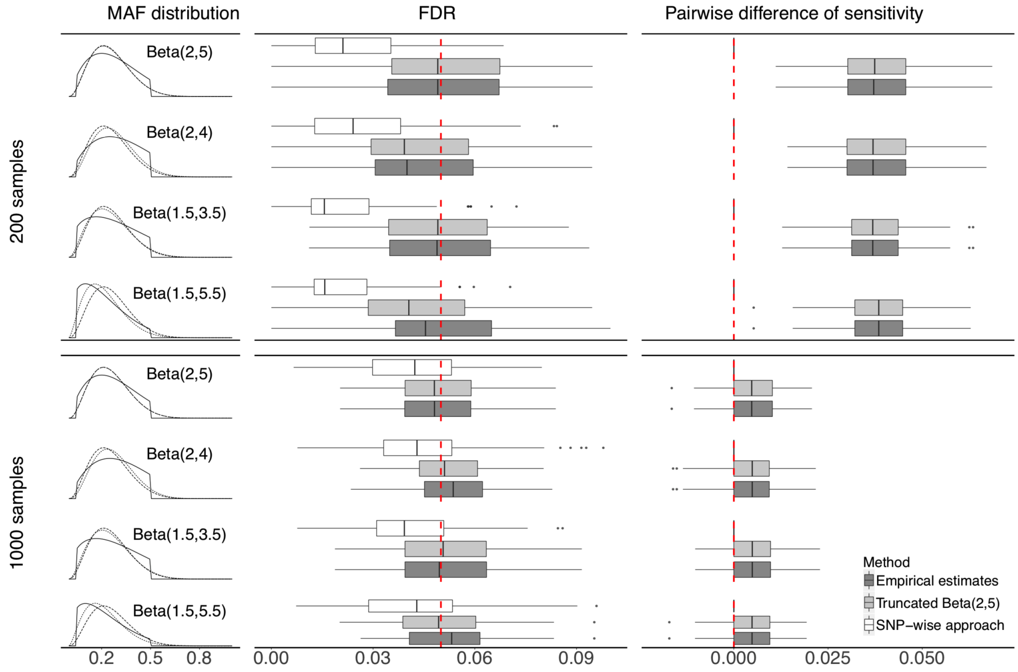

Figure S2: Simulation results for 20000 total SNPs with 200 effective SNPs.

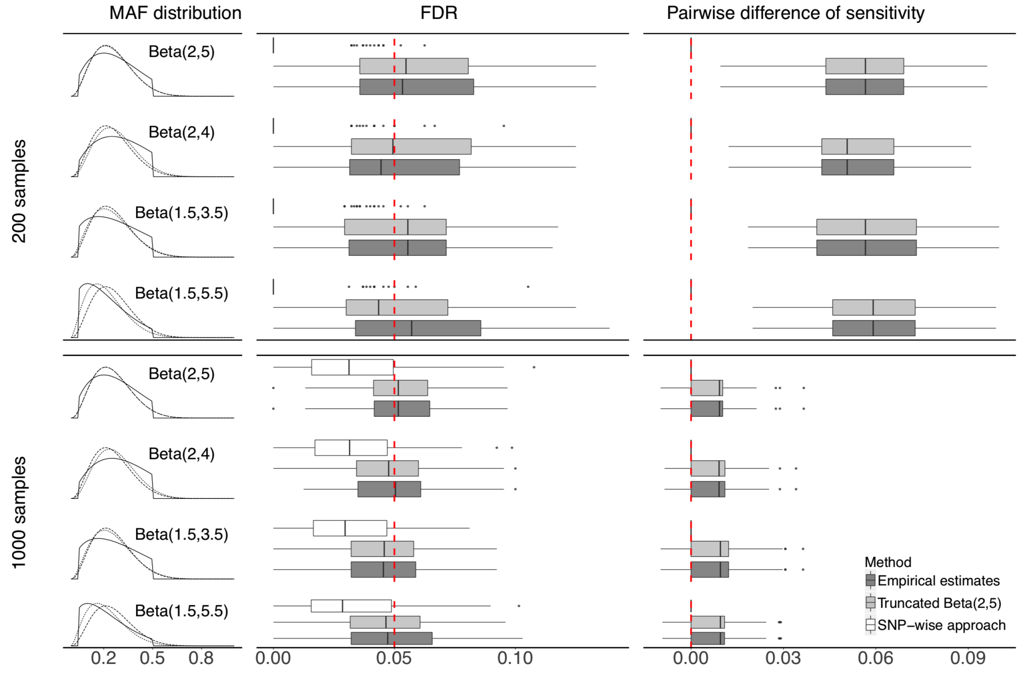

Figure S3: Boxplots for 500000 total SNPs with 100 effective SNPs.

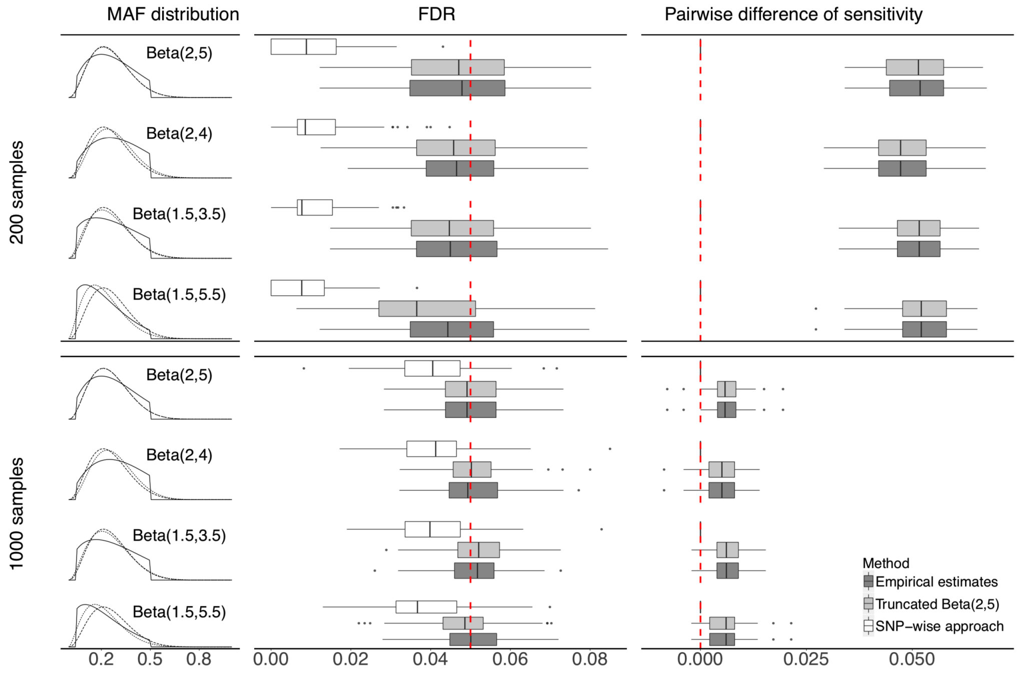

Figure S4: Boxplots for 500000 total SNPs with 500 effective SNPs.

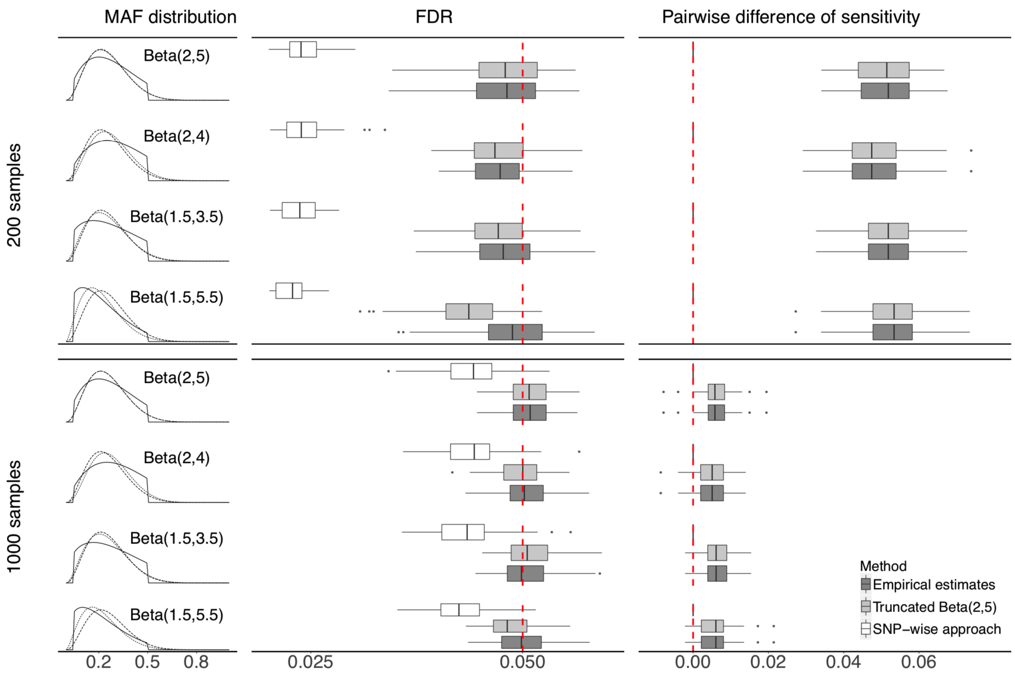

Figure S5: Simulation results for 500000 total SNPs with 5000 effective SNPs.

I Supplementary Tables

# SNPs	True π	# expected effective SNPs	MAF prior (data generation)
1000	$(0.8, 0.1, 0.1)$	200	$Beta(2,5)$
1000	$(0.8, 0.1, 0.1)$	200	$Beta(2,4)$
1000	$(0.8, 0.1, 0.1)$	200	$Beta(1.5, 3.5)$
1000	$(0.8, 0.1, 0.1)$	200	$Beta(1.5, 5.5)$
20000	$(0.99, 0.005, 0.005)$	200	$Beta(2,5)$
20000	$(0.99, 0.005, 0.005)$	200	$Beta(2,4)$
20000	$(0.99, 0.005, 0.005)$	200	$Beta(1.5, 3.5)$
20000	$(0.99, 0.005, 0.005)$	200	$Beta(1.5, 5.5)$
500000	$(0.99, 0.005, 0.005)$	5000	$Beta(2,5)$
500000	$(0.99, 0.005, 0.005)$	5000	$Beta(2,4)$
500000	$(0.99, 0.005, 0.005)$	5000	$Beta(1.5, 3.5)$
500000	$(0.99, 0.005, 0.005)$	5000	$Beta(1.5, 5.5)$
500000	$(0.999, 0.0005, 0.0005)$	500	$Beta(2,5)$
500000	$(0.999, 0.0005, 0.0005)$	500	$Beta(2,4)$
500000	$(0.999, 0.0005, 0.0005)$	500	$Beta(1.5, 3.5)$
500000	$(0.999, 0.0005, 0.0005)$	500	$Beta(1.5, 5.5)$
500000	$(0.9996, 0.0002, 0.0002)$	200	$Beta(2,5)$
500000	$(0.9996, 0.0002, 0.0002)$	200	$Beta(2,4)$
500000	$(0.9996, 0.0002, 0.0002)$	200	$Beta(1.5, 3.5)$
500000	$(0.9996, 0.0002, 0.0002)$	200	$Beta(1.5, 5.5)$
500000	$(0.9998, 0.0001, 0.0001)$	100	$Beta(2,5)$
500000	$(0.9998, 0.0001, 0.0001)$	100	$Beta(2,4)$
500000	$(0.9998, 0.0001, 0.0001)$	100	$Beta(1.5, 3.5)$
500000	$(0.9998, 0.0001, 0.0001)$	100	$Beta(1.5, 5.5)$

Table S1: Different settings to generate data for both 200-sample and 1000-sample simulations. In data analysis, for prior MAF distribution, we used both truncated Beta(2,5) and empirical distribution estimated from MAFs of all SNPs in each simulated data. Note all these prior MAF distributions used for data analysis were approximated by a Beta distribution via the moment matching. Truncated Beta(2,5) was approximated by Beta(3.29,9.56).

SNP size	Number of effective SNPs	Sample size	MAF distribution	Truncated Beta(2,5)		Empirical distribution	
				Sensitivity	|FDR-0.05|	Sensitivity	|FDR-0.05|
1000	200	200	Beta(2,5)	3.95E-18	4.69E-06	3.95E-18	1.35E-05
			Beta(2,4)	3.95E-18	0.00120716	3.95E-18	0.000328991
			Beta(1.5,3.5)	5.78E-18	0.000574269	5.78E-18	0.000405743
			Beta(1.5,5.5)	8.72E-18	5.03E-07	3.96E-18	2.30E-07
		1000	Beta(2,5)	1.19E-14	0.006444015	5.22E-15	0.020190552
			Beta(2,4)	9.19E-14	3.98E-06	4.61E-14	2.00E-05
			Beta(1.5,3.5)	2.37E-12	0.00059534	9.23E-13	0.000451012
			Beta(1.5,5.5)	9.30E-12	3.77E-05	1.65E-13	4.93E-05
20000	200	200	Beta(2,5)	3.95E-18	3.09E-06	3.95E-18	4.95E-06
			Beta(2,4)	3.96E-18	4.33E-05	3.95E-18	4.02E-05
			Beta(1.5,3.5)	3.95E-18	4.22E-10	3.95E-18	7.45E-10
			Beta(1.5,5.5)	3.95E-18	3.17E-10	3.95E-18	1.75E-09
		1000	Beta(2,5)	4.98E-08	0.002025566	2.87E-08	0.001796918
			Beta(2,4)	1.50E-05	0.000182112	1.09E-05	0.001855273
			Beta(1.5,3.5)	3.22E-07	0.000241596	5.11E-07	0.001334351
			Beta(1.5,5.5)	5.22E-07	0.00967784	2.20E-09	0.018882143
500000	100	200	Beta(2,5)	3.95E-18	2.44E-05	3.95E-18	1.72E-05
			Beta(2,4)	3.95E-18	2.12E-06	3.95E-18	3.85E-07
			Beta(1.5,3.5)	3.95E-18	2.44E-10	3.95E-18	8.44E-10
			Beta(1.5,5.5)	3.95E-18	9.16E-08	3.95E-18	5.25E-06
		1000	Beta(2,5)	7.10E-09	1.84E-06	9.28E-09	1.70E-06
			Beta(2,4)	1.35E-07	0.000379116	1.01E-07	0.000140369
			Beta(1.5,3.5)	1.03E-09	1.26E-05	5.14E-10	7.08E-05
			Beta(1.5,5.5)	1.45E-09	3.10E-05	5.71E-12	8.65E-06
500000	200	200	Beta(2,5)	3.96E-18	2.49E-12	3.96E-18	1.73E-12
			Beta(2,4)	3.95E-18	1.11E-11	3.96E-18	1.37E-11
			Beta(1.5,3.5)	3.95E-18	1.50E-13	3.95E-18	2.32E-13
			Beta(1.5,5.5)	3.96E-18	1.50E-15	3.95E-18	4.26E-14
		1000	Beta(2,5)	5.21E-13	0.000876537	5.28E-13	0.000832983
			Beta(2,4)	3.21E-09	0.002847935	1.34E-10	0.001862889
			Beta(1.5,3.5)	1.32E-14	1.81E-05	2.14E-14	5.64E-06
			Beta(1.5,5.5)	3.02E-12	0.009200061	5.07E-14	0.003422523
500000	500	200	Beta(2,5)	3.96E-18	8.91E-18	3.96E-18	9.46E-18
			Beta(2,4)	3.96E-18	9.67E-17	3.96E-18	6.08E-17
			Beta(1.5,3.5)	3.96E-18	9.75E-18	3.96E-18	1.27E-17
			Beta(1.5,5.5)	3.96E-18	4.46E-18	3.96E-18	1.88E-17
		1000	Beta(2,5)	4.42E-16	2.67E-05	4.42E-16	2.55E-05
			Beta(2,4)	2.58E-14	2.91E-07	2.41E-15	9.15E-07
			Beta(1.5,3.5)	4.83E-17	4.62E-06	4.00E-17	1.00E-06
			Beta(1.5,5.5)	1.61E-16	1.25E-08	3.87E-17	1.58E-09
500000	5000	200	Beta(2,5)	3.96E-18	8.91E-18	3.96E-18	9.46E-18
			Beta(2,4)	3.96E-18	9.67E-17	3.96E-18	6.08E-17
			Beta(1.5,3.5)	3.96E-18	9.75E-18	3.96E-18	1.27E-17
			Beta(1.5,5.5)	3.96E-18	4.46E-18	3.96E-18	1.88E-17
		1000	Beta(2,5)	4.42E-16	2.67E-05	4.42E-16	2.55E-05
			Beta(2,4)	2.58E-14	2.91E-07	2.41E-15	9.15E-07
			Beta(1.5,3.5)	4.83E-17	4.62E-06	4.00E-17	1.00E-06
			Beta(1.5,5.5)	1.61E-16	1.25E-08	3.87E-17	1.58E-09

Table S2. P-values from Wilcoxon signed rank tests between our method and the SNP-wise approach on sensitivity and $|FDR - 0.05|$ for all settings of our simulation studies.

References

Nadarajah, S and Kotz. S. (2006). R Programs for Computing Truncated Distributions. *Journal of Statistical Software* **16,** 1–8.

Yuan, M and Kendziorski. C. (2006). A unified approach for simultaneous gene clustering and differential expression identification. *Biometrics* **62,** 1089–1098.
