# Supplementary material for: Model-based clustering for identifying disease-associated SNPs in case-control genome-wide association studies
Source: Sci Rep. 2019 Sep 23;9:13686. doi: 10.1038/s41598-019-50229-6 (PMC6757104; doi:10.1038/s41598-019-50229-6)
Supplement: Supplementary file 2 — Supplementary table 3 [file 41598_2019_50229_MOESM2_ESM.pdf]

Supplementary Table S3 for "Model-based clustering for identifying disease-associated SNPs in case-control genome-wide association studies"

Yan Xu [1,+], Li Xing[1,+], Jessica Su[2], Xuekui Zhang[1,*] and Weiliang Qiu[2]
[1]: Department of Mathematics and Statistics, University of Victoria, 3800 Finnerty Road, Victoria, BC V8P 5C2 Canada
[2]: Channing Division of Network Medicine, Brigham and Women's Hospital/Harvard Medical School, 181 Longwood Avenue, Boston, MA 02115 USA
+: The authors wish it to be known that, in their opinion, the first two authors should be regarded as Joint First Authors
*: To whom correspondence should be addressed. Email: Xuekui@UVic.ca

Additional information on datasets GSE66903 and GSE65777.

Table S3(a). The top 1000 ranked SNPs in the discovery dataset with smallest raw P-values.

SNP.ID	raw p-value in GSE6	rank in GSE65777	MAF difference in G	raw p-value in GSE6	rank in GSE66903	MAF difference in G	of MAF difference?
rs10862339	5.07E-07	1	0.171349484	0.932730067	229649	-0.005767963	no
rs1344016	2.62E-06	2	0.160517826	0.866280441	212732	0.011148649	yes
rs2414277	6.31E-06	3	-0.11800904	0.480845655	113926	0.002276423	no
rs2839629	7.81E-06	4	0.093538114	0.01734138	3164	0.110135135	yes
rs4776196	9.51E-06	5	-0.121193754	0.487572911	115656	0.002276423	no
rs4145152	1.02E-05	6	-0.1525551	0.583220076	139939	-0.041738234	yes
rs4351714	1.16E-05	7	0.140034929	0.460450429	108973	-0.049430894	no
rs7597767	1.53E-05	8	0.138319293	0.964177254	238048	-0.003040541	no
rs11630101	1.68E-05	9	-0.098541981	0.698357419	169324	0.02739726	no
rs4265226	1.73E-05	10	0.144082597	0.936530741	230750	0.004878049	yes
rs4950946	1.81E-05	11	0.136850216	0.326827479	75503	-0.066554054	no
rs4245711	1.81E-05	12	0.136850216	0.272921242	62346	-0.075	no
rs10920474	1.81E-05	13	0.136850216	0.401743193	94169	-0.056097561	no
rs12140729	1.81E-05	14	0.136850216	0.511177323	121591	-0.043902439	no
rs472070	2.30E-05	15	0.138906121	0.990316296	244935	-0.000813008	no
rs7305940	2.40E-05	16	0.114444216	0.255144448	58119	0.071666667	yes
rs2068390	2.49E-05	17	-0.136922129	0.887178996	218107	-0.008455285	yes
rs186275	2.58E-05	18	0.069717024	0.298266693	68443	-0.070833333	no
rs2249235	2.66E-05	19	0.141260805	0.629544115	151892	0.031382114	yes
rs3890710	2.69E-05	20	0.139141155	0.936530741	230758	0.004878049	yes
rs1772594	2.87E-05	21	0.112851859	0.061598923	12563	-0.070894309	no
rs6472612	2.91E-05	22	0.139223341	0.936530741	230760	0.004878049	yes
rs10746192	3.55E-05	23	-0.143827543	0.766278759	186818	-0.019837398	yes
rs1527812	3.62E-05	24	-0.139451716	0.240896949	54692	-0.075121951	yes
rs2820644	3.84E-05	25	-0.136839942	0.057367204	11599	0.131666667	no
rs6431547	4.29E-05	26	0.130397292	0.974481913	240725	0.002142386	yes
rs307129	5.05E-05	27	0.032107323	0.324749473	74993	-0.068617886	no
rs10430757	5.12E-05	28	0.135062667	0.976442407	241241	-0.00195122	no
rs197687	5.24E-05	29	0.132648449	0.791280273	193309	0.01804878	yes
rs991264	5.43E-05	30	0.138452846	0.425213039	100071	0.054634146	yes
rs4973898	5.76E-05	31	0.135833162	0.206300449	46530	-0.077235772	no
rs9848083	5.95E-05	32	0.134158619	0.239195407	54406	-0.072669562	no
rs11714912	6.08E-05	33	0.135979835	0.285978211	65563	-0.06504065	no
rs2400955	6.56E-05	34	-0.135329772	0.441200519	103907	-0.052364865	yes
rs321446	6.89E-05	35	-0.126145469	0.12906255	27977	0.103424658	no
rs657642	6.93E-05	36	0.132042326	0.570946857	136686	0.037886179	yes
rs2249244	7.40E-05	37	0.0968872	0.85239951	209212	0.012357724	yes
rs2592823	7.42E-05	38	-0.132833368	0.452289844	106843	0.05496393	no
rs2629396	7.45E-05	39	-0.136018081	0.376886694	88006	-0.059674797	yes
rs7631893	7.50E-05	40	0.134240805	0.249323086	56707	-0.070569106	no
rs7625736	7.50E-05	41	0.134240805	0.249323086	56708	-0.070569106	no
rs2048177	7.75E-05	42	-0.128590507	0.949742906	234141	-0.003811504	yes
rs2252647	7.87E-05	43	0.09047668	0.85239951	209207	0.012357724	yes
rs321440	7.89E-05	44	-0.124553113	0.09622979	20610	0.10699187	no
rs6727419	8.17E-05	45	-0.127830503	0.481805016	114196	0.043055556	no
rs2459168	9.70E-05	46	0.127748099	0.847240505	207903	0.011700725	yes
rs2249236	9.70E-05	47	0.127748099	0.878075939	215740	0.009268293	yes
rs9394988	9.94E-05	48	0.135483871	0.962837696	237537	-0.003089431	no
rs2259541	0.000102148	49	0.12284775	0.356649802	82972	-0.063346883	no
rs7648952	0.000104321	50	0.129299363	0.222187025	50318	-0.07498352	no
rs9332450	0.000104321	51	0.129299363	0.268152369	61240	-0.068581081	no
rs192392	0.000105597	52	0.082473803	0.702529666	170273	-0.025365854	no
rs897117	0.000108558	53	0.129340456	0.239195407	54405	-0.072669562	no
rs1943620	0.000109161	54	0.131508116	0.992063786	245488	-0.000650407	no
rs2276868	0.000110368	55	0.129382598	0.322055645	74327	-0.061850312	no
rs2725580	0.000119307	56	0.130532155	0.719800171	174889	0.024060646	yes
rs594544	0.000121642	57	0.121815684	0.42381925	99697	-0.05025641	no
rs7921481	0.000122217	58	0.123584749	0.730965033	177796	-0.020929466	no
rs6544414	0.000126717	59	-0.124018903	0.583225644	139948	0.033658537	no
rs12706912	0.000127834	60	0.127347442	0.825667965	202120	0.014634146	yes
rs10742570	0.00012853	61	0.100113006	0.763071625	186024	0.020162602	yes
rs11577194	0.000137747	62	0.048787754	0.136293416	29649	0.00952876	yes
rs3106740	0.000140564	63	0.113740695	0.85239951	209250	0.012357724	yes
rs2459164	0.000144107	64	0.125344155	0.877727921	215652	-0.009756098	no
rs1332190	0.000166317	65	0.127871379	0.912410638	224503	-0.007317073	no
rs2450148	0.000167787	66	0.125385248	0.877727921	215644	-0.009756098	no
rs33786	0.000169304	67	-0.119395932	0.427450567	100578	-0.016585366	yes

rs4694508	0.00017587	68	-0.021686871	0.510419036	121425	-0.046143705	yes
rs16835902	0.000176263	69	-0.12694101	0.441328908	103919	0.051666667	no
rs569078	0.000176398	70	0.118111773	0.400548308	93862	-0.049593496	no
rs11717036	0.000183179	71	0.127912472	0.206300449	46535	-0.077235772	no
rs3783398	0.000184523	72	-0.123879213	0.862157764	211818	-0.011371127	yes
rs12882130	0.000185232	73	-0.125405794	0.739320459	179906	-0.022926829	yes
rs7195507	0.000188688	74	0.123831582	0.3092215	71184	0.065202703	yes
rs10501246	0.000191284	75	0.103338812	0.841988897	206520	0.013495935	yes
rs1821693	0.000192488	76	0.06005753	0.852119087	209116	0.013183916	yes
rs6820459	0.000195926	77	-0.119858229	0.81071916	198284	-0.016260163	yes
rs2065015	0.000196147	78	-0.122714198	0.755316247	183969	-0.020650407	yes
rs12894275	0.000196147	79	-0.122714198	0.830050391	203313	-0.01398374	yes
rs3783397	0.000196147	80	-0.122714198	0.830050391	203316	-0.01398374	yes
rs2065018	0.000196147	81	-0.122714198	0.755316247	183973	-0.020650407	yes
rs12879663	0.000196147	82	-0.122714198	0.755316247	183995	-0.020650407	yes
rs11109108	0.000198403	83	0.118466714	0.296297952	67911	0.072520325	yes
rs6971996	0.000210915	84	-0.12214128	0.679100895	164506	-0.029166667	yes
rs561470	0.000214864	85	0.096928293	0.500751609	118960	0.046666667	yes
rs1807939	0.000224725	86	0.058465174	0.871354919	214075	0.011382114	yes
rs2493766	0.000232666	87	-0.123772344	0.351210656	81565	0.060325203	no
rs6431549	0.000233688	88	0.128652147	0.992865983	245589	0.000668226	yes
rs28288	0.000241754	89	-0.121779179	0.426715644	100459	-0.01722973	yes
rs12585898	0.000242579	90	0.122036162	0.617392365	148708	-0.035431773	no
rs10778826	0.000244512	91	-0.125118143	0.943034756	232560	0.005067568	no
rs4239006	0.000246293	92	0.127224163	0.840167487	206006	0.014471545	yes
rs1109794	0.000250482	93	0.123464146	0.971401485	239898	0.002439024	yes
rs1772580	0.000254909	94	0.05528046	0.350463992	81406	-0.035447154	no
rs1841946	0.000262838	95	0.100154099	0.643380121	155422	0.033495935	yes
rs10862348	0.00026541	96	-0.122756975	0.962168444	237343	0.003333333	no
rs2166577	0.000266501	97	-0.121727964	0.464860486	109886	0.051219512	no
rs2123999	0.000274444	98	0.124462366	0.206300449	46533	-0.077235772	no
rs9919410	0.000276736	99	0.116272858	0.724968927	176196	-0.020929466	no
rs9923605	0.000280516	100	0.12174851	0.626721883	151059	-0.031641397	no
rs4721036	0.000282025	101	-0.113160058	0.954788635	235587	0.003739837	no
rs41331449	0.000286386	102	-0.118995274	0.713294923	173057	0.024715447	no
rs12451788	0.000288578	103	0.125713992	0.943532971	232674	-0.004677581	no
rs2209373	0.000291841	104	-0.119159647	0.662463841	160307	-0.030731707	yes
rs2246452	0.000292349	105	0.121763449	0.443368639	104601	0.049604483	yes
rs1799547	0.000296241	106	-0.121974522	0.240896949	54685	-0.075121951	yes
rs9375150	0.000296568	107	0.100154099	0.444963456	104968	0.053333333	yes
rs12545037	0.000298401	108	0.120601876	0.500568498	118888	-0.046178862	no
rs9604156	0.000304273	109	0.116683789	0.536554418	128013	-0.042926829	no
rs2223026	0.000305929	110	0.114803781	0.504493318	119980	0.041463415	yes
rs10784637	0.000307487	111	0.124167783	0.987756743	244062	-0.001002339	no
rs10156555	0.00030879	112	0.117752477	0.509624697	121331	-0.043836519	no
rs4559153	0.000311952	113	0.111084857	0.115849003	24997	-0.096097561	no
rs7311936	0.000313934	114	0.000560143	0.336079673	77790	-0.063333333	no
rs2256456	0.000314182	115	0.114495182	0.850484167	208791	0.012842466	yes
rs10510708	0.000314888	116	0.121419766	0.222187025	50316	-0.07498352	no
rs6599094	0.000314888	117	0.121419766	0.222187025	50317	-0.07498352	no
rs9842564	0.000314888	118	0.121419766	0.206300449	46532	-0.077235772	no
rs7096499	0.000316616	119	0.115407606	0.619852351	149246	-0.029430894	no
rs8130292	0.000324409	120	-0.079264145	0.135874483	29522	-0.045773471	yes
rs6801859	0.000329623	121	0.117947401	0.339491924	78611	-0.058373984	no
rs2863147	0.000335557	122	0.123645463	0.863820353	212199	-0.010894309	no
rs1396133	0.000340103	123	-0.11489472	0.583225644	139952	0.033658537	no
rs2978632	0.000340934	124	0.119786316	0.51241498	121835	0.040325203	yes
rs2479753	0.000342735	125	0.113211424	0.422445774	99326	-0.05471325	no
rs1556408	0.00034973	126	-0.120669817	0.360451378	83930	0.060325203	no
rs2850303	0.000350217	127	-0.122827204	0.194621606	43709	-0.053170732	yes
rs2819962	0.000351078	128	-0.12039871	0.32658753	75445	0.066438356	no
rs6991826	0.000354318	129	0.12422962	0.414779246	97452	-0.056260163	no
rs4961688	0.000358415	130	0.118179944	0.291499784	66834	0.076747967	yes
rs1078814	0.000359549	131	0.118814465	0.371307982	86697	-0.0633144	no
rs671576	0.00036151	132	-0.077707006	0.247947013	56394	-0.022113821	yes
rs10905680	0.00036156	133	-0.12442953	0.66252868	160326	-0.029166667	yes
rs931406	0.000366952	134	0.110120581	0.32817658	75844	-0.06046431	no
rs1263978	0.000368638	135	-0.06530969	0.66252868	160324	-0.029166667	yes
rs6499221	0.000368799	136	-0.114711321	0.363384076	84724	0.061463415	no

rs170916	0.000375362	137	-0.116716479	0.410227882	96255	-0.058699187	yes
rs16857010	0.000379109	138	-0.118502157	0.464860486	109872	0.051219512	no
rs12885387	0.000379553	139	-0.010233948	0.602593485	144885	0.034471545	no
rs307132	0.000381823	140	0.087374152	0.215275716	48645	-0.086341463	no
rs2725586	0.000386934	141	0.108933002	0.709391345	172270	0.026829268	yes
rs12910340	0.000387491	142	0.109790425	0.619069777	149028	-0.03300813	no
rs2178081	0.000392444	143	-0.120464352	0.292414453	67141	-0.066743573	yes
rs11774243	0.00039683	144	0.117793302	0.976442407	241235	-0.00195122	no
rs4812435	0.000400521	145	-0.115558897	0.845811931	207497	-0.0125	yes
rs4845406	0.000400843	146	-0.104732783	0.747328329	181919	0.022276423	no
rs10909940	0.000401592	147	-0.118698394	0.539341441	128857	0.038572459	no
rs57922	0.00041363	148	-0.082484076	0.945762359	233023	-0.004390244	yes
rs13235812	0.000414456	149	0.116945157	0.573830669	137306	0.039186992	yes
rs10869291	0.000415677	150	0.117509119	0.707707932	171632	-0.023611111	no
rs12515448	0.000419706	151	-0.124255188	0.562551484	134467	0.014471545	no
rs1799548	0.000423862	152	-0.11907746	0.302204943	69501	-0.066743573	yes
rs11244391	0.000428269	153	0.125755085	0.221466648	50181	-0.075121951	no
rs10458778	0.000428269	154	0.125755085	0.129371414	28040	-0.096327096	no
rs4486617	0.000433781	155	0.11211705	0.380027011	88849	-0.055	no
rs4731643	0.000437211	156	0.071470063	0.901359263	221703	-0.008333333	no
rs10869294	0.000440593	157	0.119675352	0.820553869	200790	0.014796748	yes
rs2258676	0.000442687	158	0.111701253	0.403342166	94466	-0.057350033	no
rs1841945	0.0004438	159	0.117464557	0.777144239	189721	0.019186992	yes
rs10447299	0.000445559	160	-0.121111568	0.187454859	41925	-0.053170732	yes
rs10781172	0.000449552	161	0.120032874	0.332299793	76916	0.063577236	yes
rs11161475	0.00045199	162	-0.121481405	0.844885296	207354	-0.014146341	yes
rs6745177	0.000452043	163	-0.114136018	0.813652091	199047	0.014189189	no
rs7588173	0.000452043	164	-0.114136018	0.916500155	225440	0.00626236	no
rs7914753	0.000452158	165	0.121588089	0.465279834	109975	0.054391892	yes
rs996261	0.000455141	166	0.114608588	0.763071625	186017	0.020162602	yes
rs2737215	0.00045572	167	-0.121808209	0.158158459	34941	-0.096242584	yes
rs4689424	0.000460493	168	0.111331416	0.699577966	169765	-0.023902439	no
rs12440578	0.000464359	169	0.11086262	0.926058842	228208	-0.005932762	no
rs2716129	0.000466083	170	0.100195192	0.890183362	218855	0.009105691	yes
rs11736183	0.000476038	171	0.113053102	0.888220405	218367	-0.009146341	no
rs3007242	0.000480838	172	-0.05047257	0.1951032	43808	0.061138211	no
rs6833000	0.000485388	173	-0.120217793	0.322741861	74480	-0.068455285	yes
rs10793993	0.000486532	174	0.124569721	0.121009912	26063	-0.096327096	no
rs41531449	0.000486729	175	0.113263939	0.920337173	226389	0.006427159	yes
rs2680296	0.000490429	176	-0.032833368	0.07396544	15323	-0.077560976	yes
rs6021602	0.000491767	177	-0.11175638	0.467817662	110678	0.051282051	no
rs10178695	0.000500661	178	-0.117320731	0.673850584	163161	-0.029166667	yes
rs7155822	0.000501076	179	-0.112903226	0.830050391	203328	-0.01398374	yes
rs11683782	0.000506068	180	0.114197658	0.486006175	115198	-0.048336798	no
rs7476580	0.000509628	181	-0.11442367	0.067187971	13800	0.121300813	no
rs12355784	0.000511268	182	-0.120988288	0.762554379	185856	0.021138211	no
rs10048993	0.000517097	183	0.112229022	0.865474842	212585	0.01039501	yes
rs2667634	0.000522813	184	0.11587138	0.687696028	166605	0.025853659	yes
rs7008284	0.000523221	185	0.109262121	0.935227252	230364	-0.005512863	no
rs2135999	0.00052373	186	-0.112625848	0.675261274	163528	0.025853659	no
rs1114876	0.000524342	187	0.124162729	0.221466648	50180	-0.075121951	no
rs7187733	0.000528731	188	0.113416889	0.750768783	182810	-0.020650407	no
rs6817116	0.000536534	189	0.113720017	0.928831513	228867	-0.005767963	no
rs874869	0.000536543	190	0.026139466	0.863352128	212102	-0.011666667	no
rs1263984	0.000540499	191	-0.066391838	0.894184379	219863	-0.008780488	yes
rs7954777	0.000551471	192	0.122364907	0.070816396	14561	-0.118733062	no
rs188642	0.000559039	193	0.090599959	0.159499299	35239	-0.098536585	no
rs7186604	0.000561256	194	0.115296897	0.70277315	170386	-0.024884641	no
rs2974293	0.000561992	195	0.000801181	0.745902882	181582	-0.023252033	no
rs4600124	0.000564474	196	-0.068349393	0.269901921	61698	0.054471545	no
rs6449584	0.000564656	197	-0.12127594	0.599149181	143926	-0.036422764	yes
rs13107487	0.000567714	198	-0.037651531	0.076742641	15923	0.016666667	no
rs12053069	0.000570914	199	-0.11160668	0.761220772	185491	0.01964672	no
rs997132	0.000571862	200	0.108475447	0.346533261	80503	0.059186992	yes
rs9577281	0.000573398	201	0.111320208	0.587747267	141097	-0.037398374	no
rs1396134	0.000575838	202	-0.112461475	0.675261274	163537	0.025853659	no
rs3999089	0.00057589	203	-0.124255188	0.762554379	185848	0.021138211	no
rs1379908	0.000579667	204	0.117437906	0.668159752	161777	0.028130081	yes
rs1927411	0.000589829	205	0.108434354	0.296438203	67946	0.065853659	yes

rs2798370	0.000590195	206	-0.111340888	0.605353876	145430	0.032682927	no
rs1388360	0.000591324	207	0.117999835	0.276592371	63305	0.070243902	yes
rs4723836	0.000592997	208	0.016858434	0.79072174	193159	0.01878708	yes
rs4676447	0.000592998	209	-0.118971793	0.568728389	136105	-0.039759439	yes
rs12713379	0.000594985	210	0.115296897	0.198800883	44736	-0.0975	no
rs7685220	0.000597125	211	0.113416889	0.443512451	104620	0.051382114	yes
rs1909915	0.000597193	212	0.112747125	0.983488339	243048	-0.001388889	no
rs7119055	0.000601254	213	0.121019108	0.985502355	243527	0.001153593	yes
rs6427765	0.000607304	214	0.111290323	0.391930689	91761	0.055	yes
rs11244392	0.000609851	215	0.122529279	0.221466648	50175	-0.075121951	no
rs41485553	0.000618304	216	-0.118625437	0.020865262	3890	-0.146901709	yes
rs12712806	0.000622321	217	0.074594206	0.686307382	166207	-0.026666667	no
rs10862342	0.000624029	218	-0.115839145	0.962168444	237336	0.003333333	no
rs1482179	0.00063584	219	-0.114705368	0.925512315	228071	0.006944444	no
rs4319778	0.000637747	220	0.112687487	0.900179234	221422	0.00796748	yes
rs6479909	0.000639361	221	0.11492706	0.405070549	94929	0.055772358	yes
rs8134673	0.000643405	222	-0.116416684	0.014339415	2515	-0.093050451	yes
rs1946601	0.000644555	223	0.107685723	0.214684539	48490	-0.085530653	no
rs949865	0.000648062	224	-0.11476024	0.995949293	246187	0.000325203	no
rs589345	0.000655629	225	0.074553113	0.080785689	16902	-0.077290705	no
rs1263981	0.000658652	226	-0.063252517	0.60716105	145898	-0.034308943	yes
rs4687494	0.000659497	227	-0.047205671	0.318219939	73420	0.067567568	no
rs6746529	0.000660373	228	0.115584549	0.26456126	60353	-0.08195122	no
rs33794	0.000663389	229	-0.104885087	0.285550609	65485	-0.052090844	yes
rs6945776	0.000664905	230	-0.114536676	0.058827597	11917	0.013348715	no
rs10818963	0.000667007	231	0.117012533	0.021928223	4139	-0.158373984	no
rs203422	0.000670959	232	-0.109852065	0.960761349	236942	-0.00313118	yes
rs17685535	0.000673713	233	0.104619902	0.32817658	75843	-0.06046431	no
rs12532693	0.000678956	234	0.109048748	0.625188454	150649	0.033658537	yes
rs2026664	0.000679755	235	0.106595439	0.775176904	189035	-0.019826965	no
rs404961	0.000692542	236	0.111109966	0.258186271	58830	-0.082893869	no
rs2738502	0.000698843	237	0.113098685	0.546302058	130485	0.038175676	yes
rs4953572	0.000699882	238	-0.108465174	0.168987669	37462	-0.091707317	yes
rs7242646	0.000700314	239	-0.114906513	0.867868873	213152	-0.011056911	yes
rs1772587	0.000704019	240	0.026494761	0.201229053	45365	-0.059837398	no
rs1772588	0.000704019	241	0.026494761	0.201630325	45453	-0.06130137	no
rs10490919	0.000707516	242	-0.069663037	0.269901921	61692	0.054471545	no
rs2450153	0.000716865	243	0.102506678	0.564930036	135051	0.03495935	yes
rs10837555	0.000717574	244	0.109831518	0.763071625	186021	0.020162602	yes
rs4712556	0.000720086	245	0.117751675	0.906017978	222816	0.007580751	yes
rs1606030	0.000722189	246	-0.10854736	0.628263316	151551	-0.033333333	yes
rs872037	0.000725879	247	-0.109441134	0.583225644	139944	0.033658537	no
rs4245772	0.000729308	248	-0.109007523	0.651724951	157487	0.028015821	no
rs10138062	0.000731817	249	0.0393158	0.789077933	192785	-0.019006849	no
rs11120300	0.000732388	250	-0.118584344	0.456130924	107520	-0.048455285	yes
rs1975259	0.000735728	251	0.110314362	0.989955414	244751	0.000833333	yes
rs10848596	0.000737477	252	0.108475447	0.85346585	209551	-0.011371127	no
rs1555474	0.000738668	253	0.117018833	0.358959365	83506	0.066829268	yes
rs10152142	0.000740671	254	0.11762893	0.624402431	150482	-0.034113382	no
rs7932578	0.000747659	255	0.1055181	0.741405075	180438	-0.021478873	no
rs1684806	0.000756816	256	-0.112502568	0.938148224	231293	-0.005203252	yes
rs1020056	0.00075684	257	0.107776865	0.126183683	27335	0.100101626	yes
rs2273702	0.000763262	258	0.108105609	0.084647512	17839	0.109268293	yes
rs1108233	0.000764565	259	-0.024830491	0.309148381	71130	0.047804878	no
rs487794	0.000771644	260	-0.114012739	0.497067371	118050	0.045691057	no
rs4314660	0.000773046	261	-0.108177522	0.495535819	117605	-0.046666667	yes
rs11635950	0.00077444	262	-0.112333966	0.746912856	181833	-0.020608108	yes
rs4729233	0.000776912	263	-0.113642901	0.449588182	106063	-0.053170732	yes
rs8127441	0.000778462	264	-0.114824327	0.006019724	938	-0.120246479	yes
rs2936914	0.000783092	265	-0.115058188	0.253993869	57874	-0.07350033	yes
rs224230	0.000784377	266	0.109450685	0.397232079	93014	-0.054548451	no
rs4479769	0.00078782	267	-0.110293815	0.069339492	14267	0.126666667	no
rs7746427	0.000798781	268	0.112934046	0.128474326	27851	0.102276423	yes
rs1563829	0.000800697	269	0.108352168	0.221466648	50178	-0.075121951	no
rs2353687	0.000801033	270	-0.112009451	0.682585445	165347	0.02796748	no
rs613049	0.000810408	271	-0.117967947	0.458136895	108183	0.028345419	no
rs7556844	0.000810629	272	0.112399836	0.93682608	230831	-0.005365854	no
rs2716764	0.000812193	273	-0.104900349	0.087445432	18478	-0.119674797	yes
rs2702327	0.000812602	274	-0.110172112	0.092778413	19718	0.114471545	no

rs2820963	0.000812898	275	-0.110386275	0.753767998	183565	0.020325203	no
rs2028119	0.000813923	276	0.11341853	0.693218306	168122	0.027804878	yes
rs10763436	0.000813975	277	0.114516129	0.291533098	66840	-0.070412518	no
rs4915919	0.000814786	278	0.120978015	0.940390597	232044	0.005	yes
rs11709661	0.000815967	279	0.085781796	0.199101006	44794	-0.078536585	no
rs11852493	0.000816242	280	-0.112461475	0.995949293	246177	0.000325203	no
rs819436	0.000821267	281	0.084405211	0.914361992	224972	-0.0075	no
rs7599534	0.000821573	282	-0.108914769	0.675261274	163542	0.025853659	no
rs27201	0.00083134	283	0.013673721	0.737407748	179408	-0.023089431	no
rs4977341	0.000834803	284	-0.021563592	0.076855075	15997	-0.119674797	yes
rs6976031	0.000836523	285	-0.059245203	0.480845655	113923	0.002276423	no
rs4952460	0.000839336	286	-0.107725498	0.675261274	163536	0.025853659	no
rs6718068	0.00084122	287	-0.105444833	0.222054459	50257	-0.08437706	yes
rs1995715	0.000846359	288	-0.11335525	0.851437575	208968	-0.0125	yes
rs2933020	0.00084688	289	0.109739059	0.582259237	139694	0.034796748	yes
rs759998	0.000847982	290	0.109256215	0.767527287	187151	-0.019281477	no
rs2114706	0.000859811	291	0.116036573	0.751820731	183116	-0.02191826	no
rs2276871	0.000861742	292	0.10787456	0.342549062	79469	-0.058658237	no
rs1627682	0.000862033	293	-0.112461475	0.938148224	231296	-0.005203252	yes
rs2135996	0.000863424	294	0.113387612	0.131265734	28503	0.005273566	yes
rs28087	0.00086389	295	-0.10854736	0.29269455	67228	0.071666667	no
rs4952359	0.000870003	296	-0.107766591	0.675261274	163543	0.025853659	no
rs13261245	0.000872483	297	-0.113157676	0.489808681	116257	-0.045203252	yes
rs7780252	0.000872722	298	-0.005516745	0.339866424	78780	0.067804878	no
rs171593	0.000874843	299	-0.108424081	0.364617738	84965	0.061300813	no
rs12515718	0.000886008	300	-0.116221787	0.245739387	55907	0.027692308	no
rs9358331	0.000886192	301	0.112975139	0.120794609	26036	0.105746742	yes
rs7556747	0.00088723	302	0.11709472	0.780261821	190436	0.018457482	yes
rs1396883	0.000888267	303	0.034497637	0.057573959	11651	0.114471545	yes
rs11602318	0.000888267	304	0.034497637	0.057573959	11653	0.114471545	yes
rs1525749	0.000898546	305	0.117423464	0.91726241	225645	0.006666667	yes
rs17398064	0.000903952	306	-0.105485926	0.30966748	71337	0.062601626	no
rs3865392	0.000907157	307	0.11289292	0.167440229	37104	0.086829268	yes
rs4952502	0.000907765	308	-0.031619654	0.399983465	93706	-0.042113821	yes
rs7864543	0.000908073	309	0.104380089	0.341342117	79128	0.063577236	yes
rs17639042	0.000910676	310	-0.114824327	0.180440591	40233	-0.089756098	yes
rs7791476	0.000910806	311	-0.109677419	0.279410267	64008	-0.0725	yes
rs625979	0.000912256	312	-0.046442936	0.10496656	22467	-0.06722973	yes
rs11595422	0.000913869	313	-0.08566879	0.604001574	145081	-0.010691614	yes
rs1003140	0.000914437	314	0.10634888	0.504216867	119799	0.043739837	yes
rs6544448	0.000918063	315	-0.106297514	0.712037492	172762	0.023577236	no
rs465821	0.000922331	316	-0.108177522	0.934466163	230108	-0.005203252	yes
rs465620	0.000922331	317	-0.108177522	0.730585629	177694	-0.021666667	yes
rs7213403	0.000923172	318	0.112823404	0.622500584	150050	0.030833333	yes
rs2373269	0.00092746	319	0.108064516	0.602503626	144817	0.036090969	yes
rs10822184	0.000930666	320	-0.067989282	0.57032618	136499	0.014527027	no
rs2260897	0.000932483	321	0.103575098	0.445718695	105104	-0.0525	no
rs10781180	0.000933315	322	0.113622355	0.31763769	73295	0.065919578	yes
rs335517	0.000933516	323	-0.107564671	0.776766949	189619	-0.019674797	yes
rs207149	0.000937126	324	0.114583119	0.11303592	24295	0.105691057	yes
rs207148	0.000937126	325	0.114583119	0.11303592	24301	0.105691057	yes
rs558448	0.000939348	326	-0.113645264	0.902971057	222051	0.008471521	no
rs10861908	0.000939548	327	0.104961989	0.272094633	62239	-0.070863546	no
rs4631962	0.000940277	328	0.115790014	0.155598512	34331	-0.095121951	no
rs7525165	0.000957335	329	0.111097236	0.948047863	233595	-0.004166667	no
rs4706918	0.000957856	330	0.106441307	0.199913847	45026	0.082439024	yes
rs7691607	0.000961784	331	0.111701253	0.565752033	135275	-0.036666667	no
rs7138489	0.000964791	332	0.061934389	0.662516835	160316	-0.032705479	no
rs1772589	0.00096747	333	0.029448259	0.203789781	45923	-0.061111111	no
rs4729239	0.000976738	334	-0.109071296	0.760935338	185401	-0.02097561	yes
rs12712756	0.000983341	335	-0.107643312	0.675261274	163530	0.025853659	no
rs472112	0.000984009	336	0.069629415	0.927441781	228533	0.006756757	yes
rs1031729	0.000990457	337	0.116159852	0.811982713	198621	0.015772358	yes
rs1841396	0.000990457	338	0.116159852	0.842499728	206673	0.013333333	yes
rs7577237	0.001001349	339	-0.103811383	0.163643584	36226	-0.096224524	yes
rs2647969	0.001001834	340	-0.109193033	0.380799767	89038	0.055479452	no
rs4571217	0.001006217	341	0.105575595	0.337025983	78014	-0.058274648	no
rs6789244	0.001008177	342	-0.059940225	0.27437391	62628	-0.084047462	yes
rs6499939	0.001014532	343	0.108804191	0.673668652	163106	-0.027317073	no

rs4281952	0.001014835	344	-0.106133142	0.675261274	163544	0.025853659	no
rs9862269	0.001017204	345	0.109091843	0.665750484	161047	0.027200277	yes
rs6712352	0.001019878	346	-0.106009862	0.790152415	192991	0.016910569	no
rs7302734	0.00102025	347	-0.013245033	0.40701394	95468	-0.056623932	yes
rs9861931	0.001021833	348	0.107540579	0.46168742	109197	-0.048617886	no
rs10735630	0.001022148	349	0.112903226	0.276592371	63295	0.070243902	yes
rs9542751	0.001023314	350	0.103278173	0.741030404	180333	-0.020487805	no
rs1681738	0.001023807	351	-0.110162131	0.248651111	56546	0.076010692	no
rs3743422	0.001032642	352	0.108064516	0.515606186	122752	0.045	yes
rs2702611	0.001033294	353	-0.11376618	0.451904427	106730	0.049769281	no
rs2710121	0.001038132	354	0.110232176	0.727126882	176752	0.024715447	yes
rs9529804	0.001039266	355	0.106595439	0.933084541	229740	-0.005203252	no
rs11245048	0.001039631	356	-0.11323197	0.836688239	205040	-0.014308943	yes
rs2070780	0.001042114	357	0.074943439	0.733518267	178482	-0.022972973	no
rs6799280	0.001044241	358	-0.11547688	0.395857372	92669	0.055609756	no
rs10863711	0.00104523	359	-0.111300662	0.394179785	92251	-0.057858688	yes
rs6537651	0.001050311	360	-0.117762482	0.146395959	32070	0.099486301	no
rs11123638	0.001055416	361	-0.110322211	0.320166379	73978	0.071127503	no
rs1599386	0.001057246	362	-0.114824327	0.506915103	120667	-0.023252033	yes
rs13338014	0.001058501	363	0.107088556	0.673668652	163128	-0.027317073	no
rs10207274	0.001058583	364	-0.011641125	0.586891178	140861	0.036255768	no
rs7923313	0.001059225	365	-0.108439484	0.414477799	97405	-0.055833333	yes
rs716877	0.001063195	366	0.109748532	0.991737706	245326	0.000675676	yes
rs27908	0.001064721	367	-0.106872817	0.113686786	24486	0.106624918	no
rs33783	0.001070694	368	-0.109893847	0.697327289	169061	0.028333333	no
rs7504457	0.001071351	369	0.104797617	0.036403739	7136	-0.136824324	no
rs1078328	0.001072243	370	-0.106749538	0.19320985	43424	0.086354647	no
rs11604069	0.001072811	371	-0.010596487	0.108869047	23404	0.117316473	no
rs6977955	0.001073232	372	0.100257652	0.347589747	80716	-0.062764228	no
rs1887866	0.001075043	373	-0.108752825	0.811131879	198368	0.015820699	no
rs2643224	0.001078496	374	0.109590888	0.978857117	241964	-0.001788618	no
rs2393969	0.001081284	375	-0.116129032	0.762554379	185843	0.021138211	no
rs983826	0.001084124	376	0.056955003	0.77270568	188460	-0.005528455	no
rs1872825	0.001087261	377	0.103780563	0.894222428	219915	-0.008455285	no
rs1872826	0.001087261	378	0.103780563	0.926058842	228205	-0.005932762	no
rs1195904	0.001091215	379	0.109543867	0.356967241	83066	-0.058536585	no
rs10484349	0.001091959	380	-0.112708034	0.559411885	133702	0.037948718	no
rs1670537	0.001096581	381	0.115343259	0.811776062	198562	-0.016222062	no
rs4729281	0.001097748	382	0.112687487	0.298946921	68624	0.06872116	yes
rs13044355	0.001105626	383	-0.111434148	0.039246554	7726	-0.154166667	yes
rs11223211	0.001107488	384	-0.068029587	0.30514207	70001	0.074471545	no
rs7945025	0.001122098	385	0.102753236	0.522979871	124660	-0.040487805	no
rs9637300	0.001124616	386	0.076417991	0.568561408	136081	0.038851351	yes
rs701848	0.001132275	387	-0.110417095	0.741705953	180509	-0.024227642	yes
rs11658900	0.00113323	388	0.104396528	0.383594876	89690	-0.063224447	no
rs3914238	0.001133402	389	0.1106842	0.895736501	220206	-0.008569545	no
rs2281530	0.001134108	390	0.089333196	0.868826348	213390	0.012028065	yes
rs531071	0.001136146	391	-0.053616191	0.168229779	37332	-0.095284553	yes
rs9356730	0.00113918	392	0.109749332	0.128474326	27857	0.102276423	yes
rs7578278	0.001141999	393	-0.106009862	0.787479167	192333	0.017303889	no
rs6886562	0.001143816	394	0.085822889	0.733302013	178403	-0.023252033	no
rs11938222	0.001144626	395	0.109790786	0.468959484	111050	0.047804878	yes
rs252827	0.001145928	396	-0.102260119	0.86956549	213609	0.010833333	no
rs875390	0.001146435	397	0.053581769	0.855898133	210170	-0.012195122	no
rs2657888	0.001152695	398	0.107190002	0.240371763	54604	-0.079182412	no
rs589465	0.001155755	399	0.100412244	0.715115287	173664	0.023611111	yes
rs17206048	0.001160339	400	0.108557633	0.69916137	169671	-0.026178862	no
rs1508123	0.001168561	401	-0.105448718	0.491051822	116550	0.043027749	no
rs10233374	0.001177972	402	0.105606513	0.456875197	107744	0.053724456	yes
rs4668940	0.001179063	403	-0.112540326	0.289641834	66400	-0.071869919	yes
rs1872364	0.001181075	404	-0.111845079	0.901162539	221629	-0.008617886	yes
rs1385344	0.001181075	405	-0.111845079	0.919167677	226157	-0.007086355	yes
rs6887081	0.001181976	406	-0.043979864	0.956732442	236039	0.003414634	no
rs1162394	0.001185401	407	-0.110786932	0.566616946	135434	0.03902439	no
rs7009673	0.001186756	408	0.108552632	0.785664237	191887	-0.016809492	no
rs921038	0.001192945	409	0.105331827	0.675261274	163547	0.025853659	yes
rs11789058	0.001193946	410	0.082597082	0.867270051	212997	-0.01203252	no
rs2362747	0.001194588	411	0.105231596	0.897493344	220635	-0.008333333	no
rs1995738	0.001195507	412	-0.104581878	0.730849562	177775	0.021259064	no

rs11578753	0.001204372	413	-0.10691391	0.715571548	173806	0.023739837	no
rs12926192	0.001204848	414	0.112934046	0.293029986	67317	-0.075284553	no
rs8080915	0.001208156	415	0.090641052	0.900650927	221526	0.008943089	yes
rs13018645	0.001210648	416	0.112687487	0.582259237	139712	0.034796748	yes
rs8031905	0.001211758	417	0.103862749	0.8671494	212970	-0.010810811	no
rs7557744	0.001212346	418	0.109915219	0.948643093	233760	0.004166667	yes
rs1999975	0.001212622	419	-0.027974111	0.708839166	172008	0.001138211	no
rs12403575	0.001212881	420	-0.110036928	0.654497584	158135	-0.030308219	yes
rs9613266	0.001216876	421	-0.115029792	0.297531068	68192	-0.07203252	yes
rs11102050	0.001218558	422	-0.116159951	0.263603013	60146	0.075684932	no
rs1290627	0.00122004	423	0.106030409	0.748158912	182171	-0.020813008	no
rs1995636	0.001221215	424	0.111821086	0.127424983	27606	-0.101680949	no
rs11123639	0.001224882	425	-0.109030203	0.411386792	96524	0.057886179	no
rs1508120	0.00122779	426	-0.103928867	0.533850426	127389	0.038590043	no
rs1354982	0.001234333	427	-0.104622971	0.583225644	139945	0.033658537	no
rs489045	0.001234521	428	0.109369797	0.603177033	144973	-0.033219178	no
rs4283935	0.001236478	429	0.016899527	0.666849026	161291	0.03	yes
rs2400928	0.001245144	430	0.106451613	0.765485467	186631	-0.021025641	no
rs9577569	0.001253147	431	0.105619478	0.550516575	131441	-0.041666667	no
rs1789756	0.001254042	432	-0.110828079	0.743516335	180987	-0.023589744	yes
rs1048821	0.001259464	433	0.110532703	0.481283544	114077	-0.050428477	no
rs6959385	0.001267287	434	0.108033696	0.11964578	25819	0.105691057	yes
rs7561570	0.001267584	435	-0.104153355	0.84547957	207446	-0.01252472	yes
rs1791977	0.001268441	436	-0.110334909	0.643338771	155390	-0.034070952	yes
rs928336	0.001271436	437	-0.113098822	0.345109828	80006	0.065691057	no
rs10867644	0.001272321	438	-0.107396754	0.643066542	155360	-0.030833333	yes
rs755403	0.001272989	439	0.064998973	0.504493318	119954	0.041463415	yes
rs7534689	0.001273478	440	-0.077665913	0.457274895	107937	-0.023252033	yes
rs10933599	0.001280921	441	0.101941648	0.942509524	232487	-0.005	no
rs4720121	0.001282137	442	-0.102054654	0.024525445	4669	0.14796748	no
rs10224784	0.001283518	443	-0.08566879	0.224953724	50989	-0.066504065	yes
rs940818	0.001287873	444	-0.102260119	0.30966748	71347	0.062601626	no
rs4527516	0.001296839	445	0.101818369	0.846183869	207549	0.0125	yes
rs2701285	0.001297726	446	0.002486131	0.184735375	41322	0.0875	yes
rs2205373	0.001299999	447	0.103834064	0.47704872	112884	0.0468029	yes
rs4270392	0.001300474	448	-0.104417506	0.88806613	218291	0.009105691	no
rs7694175	0.001308168	449	0.106439739	0.810917663	198326	-0.017723577	no
rs7024033	0.001308168	450	0.103123074	0.546853081	130652	-0.037235772	no
rs1383349	0.001312075	451	0.110108897	0.107426281	23104	-0.103909121	no
rs336570	0.001320102	452	-0.113190877	0.494122579	117265	-0.023252033	yes
rs3848453	0.001325081	453	0.02323555	0.955852889	235852	0.003790376	yes
rs2923437	0.001325324	454	0.114444216	0.336864707	77975	-0.064149683	no
rs132035	0.001325414	455	0.103369632	0.992063786	245466	-0.000650407	no
rs1033491	0.001329611	456	-0.105116088	0.895879957	220279	0.009105691	no
rs10457006	0.001332513	457	0.103646354	0.707856429	171727	-0.02504065	no
rs2043608	0.001335016	458	-0.087261146	0.882915562	217023	0.010081301	no
rs7487781	0.001335016	459	-0.087261146	0.882915562	217033	0.010081301	no
rs9325410	0.001344298	460	-0.111264224	0.726579864	176614	0.001138211	no
rs2037000	0.001346743	461	0.112399836	0.791280273	193315	0.01804878	yes
rs1203382	0.001347359	462	0.096219437	0.230253965	52284	-0.077785102	no
rs4722760	0.001348845	463	0.099321964	0.298133979	68411	-0.069430894	no
rs10761779	0.001350038	464	-0.114536676	0.762554379	185844	0.021138211	no
rs10861911	0.001350946	465	0.101736182	0.236726689	53817	-0.077620303	no
rs4541599	0.001351344	466	0.111790168	0.133088193	28958	0.104243234	yes
rs7873865	0.001351794	467	0.104798556	0.614157918	147824	-0.032845528	no
rs448936	0.001352659	468	-0.105061716	0.252703043	57551	0.076923077	no
rs11731573	0.001359206	469	-0.023155948	0.738516104	179767	0.022276423	no
rs322838	0.001359797	470	0.101401628	0.012819203	2213	0.166829268	yes
rs169095	0.001362164	471	0.095742915	0.691143754	167371	0.024060646	yes
rs12700629	0.001363149	472	-0.022875817	0.027495327	5304	-0.139492148	yes
rs268950	0.001364596	473	0.10210602	0.799470273	195475	-0.017398374	no
rs10733309	0.001364678	474	0.105013355	0.124885605	27015	0.113333333	yes
rs1538467	0.001365273	475	0.076227656	0.249722716	56791	-0.083089431	no
rs932403	0.001365731	476	0.116159852	0.962851542	237557	-0.003252033	no
rs2350192	0.001367861	477	-0.111598521	0.569950114	136422	-0.039837398	yes
rs1243564	0.001367861	478	-0.111598521	0.916069619	225350	0.006829268	no
rs4835599	0.001370362	479	-0.111639614	0.280396307	64273	-0.07203252	yes
rs1599376	0.001370437	480	-0.111762893	0.626519952	150995	-0.036585366	yes
rs10761221	0.001378716	481	0.107704952	0.468040931	110741	0.043739837	yes

rs7013415	0.001379237	482	0.103092254	0.865139375	212508	-0.01203252	no
rs7902942	0.001379351	483	-0.111034603	0.324749473	74994	-0.068617886	yes
rs1449146	0.00138018	484	-0.111310869	0.766682924	186967	0.021588662	no
rs11072794	0.001381455	485	0.100443162	0.478057409	113298	0.043333333	yes
rs10821121	0.001383195	486	0.108296309	0.41171262	96634	0.049949883	yes
rs2804910	0.001384391	487	0.105884778	0.271801059	62176	0.069268293	yes
rs1610158	0.001388534	488	-0.113147069	0.67521102	163515	-0.017723577	yes
rs726833	0.001389068	489	0.04094925	0.234749672	53318	-0.077560976	no
rs2255693	0.001390022	490	0.107499486	0.630771314	152127	0.029105691	yes
rs6465535	0.001392782	491	0.110390819	0.309433047	71261	0.066829268	yes
rs7624552	0.001393538	492	0.094339429	0.895836779	220224	-0.008292683	no
rs1268418	0.001394004	493	-0.050390384	0.983888161	243176	-0.001374098	yes
rs1836649	0.001396822	494	-0.1084549	0.78790501	192410	-0.017723577	yes
rs10739887	0.001399934	495	0.096465995	0.369322817	86168	0.060325203	yes
rs7287070	0.001400264	496	0.103369632	0.692634486	167846	0.024878049	yes
rs1836650	0.00140393	497	-0.07762482	0.72186319	175482	0.023414634	no
rs1922404	0.001404117	498	-0.095264023	0.317608935	73283	-0.054308943	yes
rs329304	0.00142252	499	-0.113026505	0.181096312	40393	-0.091952055	yes
rs6495436	0.001425222	500	0.104438052	0.730509828	177636	-0.021788618	no
rs976160	0.001433249	501	-0.103536529	0.692809873	167956	0.025684932	no
rs7232649	0.001435874	502	-0.108019794	0.35392872	82204	-0.064065041	yes
rs1007452	0.001447548	503	-0.105804397	0.691419553	167486	-0.027642276	yes
rs6469440	0.001447855	504	0.096589275	0.920492483	226455	0.00601626	yes
rs10774214	0.001447908	505	0.110930758	0.078888511	16453	-0.118333333	no
rs7794063	0.001450367	506	0.105621864	0.952983705	235170	0.003739837	yes
rs140512	0.001450621	507	-0.107602219	0.668422888	161829	-0.027382646	yes
rs6885628	0.001450664	508	0.11436203	0.81759384	200047	0.015985498	yes
rs14038	0.001452809	509	0.105684885	0.503351745	119654	0.044715447	yes
rs4784170	0.001458248	510	0.112540193	0.249642206	56778	-0.08195122	no
rs12051648	0.00146013	511	0.115015974	0.678366966	164374	-0.028333333	no
rs12655690	0.00146081	512	0.110223642	0.773168303	188623	0.019186992	yes
rs2733753	0.001471302	513	0.104998454	0.80753661	197493	-0.016090786	no
rs4616689	0.001473155	514	-0.111553128	0.564179671	134928	-0.029828609	yes
rs169410	0.001481549	515	-0.107396754	0.953642047	235307	0.003577236	no
rs2140016	0.001488012	516	0.107252928	0.766608692	186942	-0.021449171	no
rs7652267	0.00148965	517	-0.023155948	0.229607492	52117	-0.089485827	yes
rs4612917	0.001496231	518	0.020755108	0.772602831	188447	0.019752662	yes
rs10234557	0.001498378	519	-0.107643312	0.924731418	227734	-0.006504065	yes
rs17098	0.001507451	520	0.007345387	0.589559072	141549	-0.036486486	no
rs4092077	0.001511525	521	-0.111466088	0.441991337	104173	0.050769231	no
rs9358376	0.001512639	522	0.109327012	0.994147858	245931	-0.000494397	no
rs7725649	0.001518867	523	0.103782001	0.156549651	34562	-0.09300813	no
rs2245221	0.001527086	524	-0.110376002	0.154621859	34038	-0.096422764	yes
rs462318	0.001529568	525	-0.104869529	0.077808389	16225	-0.117235772	yes
rs1567492	0.001537005	526	-0.102095747	0.701864434	170158	-0.027217015	yes
rs1470159	0.001540436	527	0.100102733	0.301310835	69294	0.0675	yes
rs6971826	0.001545179	528	-0.1032669	0.055135042	11079	-0.015279241	yes
rs659406	0.001558503	529	0.098752963	0.895836779	220228	0.008292683	yes
rs7619011	0.00156075	530	-0.111404053	0.566010271	135351	-0.029736051	yes
rs6575988	0.001564041	531	0.101653996	0.043483328	8603	0.132290185	yes
rs336583	0.001569992	532	-0.111598521	0.494122579	117266	-0.023252033	yes
rs336585	0.001569992	533	-0.111598521	0.494122579	117267	-0.023252033	yes
rs654534	0.001570624	534	-0.110233948	0.052016947	10423	0.009446925	no
rs2981182	0.001576701	535	-0.106698171	0.698691519	169551	0.024552846	no
rs11119107	0.001586424	536	0.103328539	0.95561649	235737	0.003739837	yes
rs1836796	0.001587488	537	-0.108742552	0.616091082	148384	-0.036585366	yes
rs2213736	0.001589397	538	0.111382782	0.012533265	2157	-0.179512195	no
rs4762652	0.001593397	539	-0.088853503	0.982949911	242912	-0.001483191	yes
rs3975315	0.001594319	540	-0.051982741	0.169102207	37532	-0.096422764	yes
rs4778846	0.001598142	541	-0.005179901	0.22041153	49926	-0.091216216	yes
rs16902303	0.001601396	542	0.103616191	0.946227133	233129	0.004280822	yes
rs2221720	0.00160196	543	0.040963031	0.056164072	11294	0.116183256	yes
rs12896331	0.001610308	544	0.066591329	0.946180234	233121	-0.004227642	no
rs10497205	0.00161345	545	-0.107889871	0.555552196	132692	-0.039674797	yes
rs9475312	0.001622995	546	-0.103883861	0.425226673	100088	0.050758075	no
rs2047420	0.001623641	547	0.102558044	0.217959226	49397	0.07902439	yes
rs4797723	0.001627837	548	-0.106698959	0.783420411	191171	-0.018333333	yes
rs11143982	0.001628369	549	-0.098705568	0.92626446	228242	-0.006164384	yes
rs16964164	0.001632732	550	0.105054448	0.249642206	56773	-0.08195122	no

rs1457536	0.001634111	551	0.029949217	0.474317619	112306	0.047297297	yes
rs1732314	0.001639666	552	-0.10577957	0.963777288	237912	0.002945253	no
rs4370045	0.001641413	553	-0.111598521	0.447316484	105417	-0.042113821	yes
rs2299253	0.001648704	554	-0.058434354	0.386270137	90390	-0.009918699	yes
rs1821943	0.001648997	555	0.098140538	0.495860479	117718	0.043739837	yes
rs7186591	0.001651557	556	0.104231926	0.622329692	150007	-0.033017211	no
rs13175935	0.001656882	557	0.105376344	0.12945	28053	0.092357724	yes
rs2647970	0.00165703	558	-0.10474625	0.504493318	119976	0.041463415	no
rs13259025	0.001658978	559	-0.107101365	0.426218889	100339	-0.053333333	yes
rs2965392	0.001660916	560	-0.055086056	0.391450631	91593	-0.059837398	yes
rs2192858	0.001661407	561	0.105824943	0.956477539	235961	0.003625577	yes
rs12965056	0.001664175	562	-0.107142857	0.596363495	143175	0.035	no
rs241333	0.001666373	563	-0.016745428	0.111800165	23974	0.104552846	no
rs12504806	0.001666906	564	0.058588453	0.118369957	25525	-0.103089431	no
rs11736360	0.001671503	565	0.107958725	0.535332649	127640	-0.039512195	no
rs10180222	0.001671949	566	-0.006617571	0.241481568	54789	-0.02100271	yes
rs12033610	0.001680268	567	-0.105598932	0.424128625	99762	-0.052702703	yes
rs2400954	0.00168242	568	-0.107273474	0.323394263	74680	-0.066341463	yes
rs6754794	0.001686997	569	-0.105658044	0.394378472	92278	0.060808553	no
rs877290	0.001687607	570	-0.050293724	0.445558807	105051	-0.048780488	yes
rs10099877	0.001687607	571	-0.050293724	0.439938688	103531	-0.05	yes
rs11138170	0.001688742	572	0.096959112	0.605866996	145597	0.033658537	yes
rs2090882	0.001688862	573	0.107335114	0.513064665	121923	-0.045203252	no
rs6720433	0.001693994	574	-0.11055419	0.676889865	163937	0.030081301	no
rs12211996	0.001694232	575	-0.107872708	0.658393166	159309	-0.028455285	yes
rs197766	0.001696052	576	0.102450775	0.769552348	187631	0.019178082	yes
rs8058005	0.001697954	577	-0.104787343	0.490005566	116332	0.04699187	no
rs2315599	0.001706013	578	-0.090384615	0.202753074	45673	0.081081081	no
rs7450440	0.001706216	579	-0.011927265	0.16238246	35941	-0.088617886	yes
rs4623490	0.001707825	580	-0.104184273	0.963521105	237823	-0.003076923	yes
rs11495981	0.001708384	581	0.096055065	0.318496273	73526	-0.066908372	no
rs6479401	0.001714335	582	0.100153032	0.542339979	129420	0.039349593	yes
rs2981174	0.001716638	583	-0.105831668	0.689104419	166995	0.025543837	no
rs1107744	0.001723762	584	0.106506239	0.594560863	142811	-0.035616438	no
rs17496473	0.001726682	585	-0.105229094	0.976342312	241198	-0.002113821	yes
rs2569343	0.001732014	586	-0.11132134	0.123912876	26748	-0.10195122	yes
rs4563279	0.001740697	587	-0.101273885	0.787479167	192329	0.017303889	no
rs6840135	0.001743037	588	-0.055086056	0.472403135	111766	-0.046341463	yes
rs11166002	0.00174364	589	0.095313922	0.718926203	174645	-0.023310811	no
rs1377495	0.001746	590	-0.005348861	0.261567393	59721	0.081081081	no
rs6973092	0.001746022	591	0.099031024	0.036373969	7127	0.150081301	yes
rs7733775	0.001747192	592	0.084230532	0.281040375	64400	-0.069756098	no
rs4974067	0.001748795	593	0.103451818	0.258406976	58867	-0.069444444	no
rs10774915	0.001750459	594	-0.10694473	0.398721751	93327	-0.056422764	yes
rs12712791	0.001760098	595	-0.108331621	0.3363285	77835	-0.065833333	yes
rs7817885	0.001760251	596	-0.112263833	0.71675543	174053	-0.024166667	yes
rs1536651	0.001761785	597	-0.104859256	0.016579648	2983	-0.11300813	yes
rs11166113	0.001764285	598	-0.035976988	0.73278317	178169	0.024552846	no
rs10430983	0.001768805	599	0.103780563	0.090163311	19149	-0.112887278	no
rs9516572	0.001770887	600	0.109502774	0.998005645	246621	-0.000164799	no
rs10778810	0.001773062	601	0.095867258	0.297595216	68224	-0.066666667	no
rs1408889	0.001773998	602	0.103625541	0.731875794	177990	0.022601626	yes
rs9577594	0.00178346	603	0.10795151	0.6928765	167977	-0.026341463	no
rs554531	0.001787062	604	-0.013367	0.708839166	172007	0.001138211	no
rs6006926	0.001788836	605	-0.101931375	0.671977143	162650	0.0275	no
rs11208285	0.001791345	606	0.10157181	0.912410638	224496	-0.007317073	no
rs6946407	0.001794225	607	0.102722416	0.213686547	48284	-0.077398374	no
rs1498770	0.001801511	608	-0.101360404	0.56816412	135908	0.036130137	no
rs11129876	0.001805845	609	0.103408572	0.319078873	73685	-0.061470007	no
rs3755130	0.001808526	610	0.106966917	0.320861322	74104	0.062837838	yes
rs4626423	0.001813501	611	-0.103640701	0.602811769	144928	0.033783784	no
rs132047	0.001814087	612	0.020040662	0.938599672	231392	-0.005438365	no
rs2893312	0.001816378	613	0.096096158	0.298133979	68405	-0.069430894	no
rs4084672	0.001816424	614	-0.108481913	0.798519025	195209	-0.017798286	yes
rs1435642	0.001823625	615	-0.101561537	0.791810616	193533	0.017948718	no
rs12526892	0.001826551	616	-0.102424491	0.807889659	197577	0.01713909	no
rs11143769	0.001839193	617	0.105033502	0.13942217	30458	0.036747967	yes
rs3750163	0.001843245	618	0.043877165	0.24251539	55110	0.086538462	yes
rs12701735	0.001854055	619	-0.107889871	0.988718331	244352	-0.001025641	yes

rs2737203	0.00185985	620	-0.105103205	0.069463704	14286	-0.126341463	yes
rs322826	0.001861728	621	0.102392784	0.019012573	3489	0.159459459	yes
rs1389603	0.001862541	622	0.045807228	0.988115557	244188	-0.00097561	no
rs975892	0.001864327	623	0.098304911	0.067391182	13864	0.115934959	yes
rs6479911	0.00186724	624	0.101975898	0.331764744	76699	0.064189189	yes
rs6075977	0.001868018	625	0.10590713	0.040329842	7933	0.142276423	yes
rs12895948	0.001872051	626	-0.108156106	0.98789712	244115	-0.001025641	yes
rs4676366	0.001883421	627	-0.102742963	0.847355111	207990	-0.012845528	yes
rs621314	0.001885192	628	0.105948223	0.071481032	14722	-0.119512195	no
rs4799915	0.001885192	629	0.105948223	0.131397976	28532	0.0975	yes
rs7543645	0.001885345	630	0.10157181	0.78577569	191913	-0.018333333	no
rs13167393	0.001887305	631	0.098551469	0.247867679	56384	-0.07183428	no
rs8036757	0.001891985	632	-0.028906984	0.028213343	5479	-0.063089431	yes
rs925760	0.001899224	633	-0.095305116	0.419111938	98499	-0.01722973	yes
rs12154758	0.001899752	634	0.005864166	0.309148381	71122	0.047804878	yes
rs8014013	0.001901135	635	0.099154901	0.084647512	17842	0.109268293	yes
rs1399130	0.001901252	636	-0.102806871	0.155138383	34242	0.016747967	no
rs2865195	0.001905977	637	0.101171153	0.537603236	128422	-0.04	no
rs10804463	0.001906103	638	-0.10456507	0.094762101	20273	-0.111282051	yes
rs11125817	0.001906735	639	-0.007109102	0.665517081	160959	0.02796748	no
rs9292581	0.001908987	640	-0.100797219	0.41774999	98269	-0.05158207	yes
rs10948926	0.001909303	641	-0.103192985	0.573330415	137188	0.03495935	no
rs2215637	0.001915708	642	-0.103308785	0.232294179	52730	0.035810811	no
rs990902	0.001916758	643	0.063681336	0.234564531	53284	0.082191781	yes
rs191985	0.001920732	644	0.100587113	0.850957418	208907	-0.011666667	no
rs2866592	0.001925343	645	0.09626053	0.129639199	28125	0.095934959	yes
rs1874537	0.001925582	646	-0.108331621	0.136561825	29750	-0.015833333	yes
rs10092220	0.001933952	647	-0.101150606	0.073935347	15306	-0.116260163	yes
rs7748788	0.001936008	648	-0.059985617	0.445135267	104986	-0.054308943	yes
rs36088	0.001937008	649	0.105397022	0.18308349	40929	0.090081301	yes
rs11050907	0.001938955	650	-0.106174235	0.952119628	234922	-0.004065041	yes
rs17658458	0.001944985	651	-0.106247919	0.521621528	124185	-0.040833333	yes
rs1435484	0.001945416	652	-0.105101515	0.785871549	191945	-0.018243243	yes
rs2249852	0.001949018	653	0.097319347	0.85083507	208899	0.011544715	yes
rs9925381	0.001951629	654	0.102270392	0.76881667	187447	-0.019446276	no
rs3740358	0.001962556	655	0.093866858	0.132117931	28742	0.108943089	yes
rs1876213	0.001963022	656	0.104890076	0.10027698	21474	-0.110472973	no
rs2251671	0.001966844	657	-0.103578467	0.008980469	1487	-0.180189673	yes
rs12464547	0.001967302	658	-0.103636737	0.672393021	162791	-0.029828609	yes
rs6843734	0.001969109	659	0.097340359	0.151386909	33237	-0.095945946	no
rs6847339	0.001970975	660	0.104314773	0.844837788	207347	0.014379764	yes
rs2850492	0.001973182	661	-0.107150195	0.486864882	115379	0.047089041	no
rs4712488	0.001973981	662	0.10483871	0.128474326	27862	0.102276423	yes
rs7308980	0.001975842	663	-0.092038217	0.317608935	73289	-0.054308943	yes
rs1408890	0.001979307	664	0.100349291	0.916283665	225424	-0.006756757	no
rs11161473	0.001985306	665	-0.106657078	0.781600562	190904	0.020267949	no
rs11258591	0.001986662	666	-0.097565235	0.89365296	219628	0.008292683	no
rs12042401	0.001987631	667	-0.111237613	0.086141497	18205	0.117501648	no
rs931382	0.001988158	668	-0.105188001	0.745432687	181427	0.021138211	no
rs730402	0.001988366	669	0.005711938	0.594480148	142763	0.034634146	yes
rs10487285	0.001990945	670	0.105537292	0.934466163	230112	-0.005203252	no
rs984551	0.001992871	671	0.066591329	0.870868042	213925	-0.01087673	no
rs234220	0.001994826	672	0.037764537	0.973128242	240377	0.002276423	yes
rs7776738	0.002001706	673	-0.043938771	0.018115614	3293	-0.146341463	yes
rs10818964	0.002001793	674	0.105198941	0.043575611	8623	-0.137112722	no
rs9656035	0.002010659	675	-0.102537497	0.619767528	149174	-0.034308943	yes
rs9650306	0.002010989	676	0.100684576	0.412868431	96950	0.054460408	yes
rs7159296	0.002015403	677	-0.1031128	0.175190425	38891	0.084102564	no
rs944779	0.002022181	678	0.013599021	0.576123776	137995	0.014471545	yes
rs7357472	0.002029834	679	-0.105804397	0.070054468	14379	-0.128873239	yes
rs2721954	0.002029834	680	-0.105804397	0.071926963	14859	-0.126071193	yes
rs1195902	0.002030338	681	0.103315215	0.358631348	83408	-0.059760274	no
rs1791644	0.002030435	682	-0.103523731	0.738006076	179574	0.021463415	no
rs1791642	0.002030435	683	-0.103523731	0.738006076	179580	0.021463415	no
rs1732311	0.002030435	684	-0.103523731	0.754936852	183867	0.020833333	no
rs2839628	0.002034529	685	0.101036662	0.111449596	23899	0.101027397	yes
rs2929111	0.002035506	686	0.023392233	0.659560919	159529	-0.018243243	no
rs2955864	0.002035506	687	0.023392233	0.661788579	160176	-0.017633487	no
rs10131946	0.002036435	688	-0.105534371	0.540571935	129075	-0.030833333	yes

rs2093256	0.002039791	689	-0.104982535	0.405187797	95006	-0.057560976	yes
rs12209943	0.002040267	690	-0.106256421	0.374301258	87320	0.056910569	no
rs33767	0.002041739	691	-0.103472365	0.8171168	199936	0.016747967	no
rs10865895	0.002047742	692	0.101859462	0.341433713	79180	-0.057235772	no
rs2669986	0.002049879	693	-0.100380111	0.923107593	227224	-0.006348146	yes
rs707137	0.002051153	694	0.103698377	0.373211543	87068	-0.0575	no
rs3929608	0.002059919	695	0.10058933	0.609480811	146580	-0.032959789	no
rs2700797	0.002062295	696	0.103297719	0.284546068	65189	0.070204351	yes
rs3737434	0.002063487	697	0.101941648	0.138415063	30187	0.092520325	yes
rs12772884	0.002065716	698	-0.106615985	0.634241637	153050	-0.035447154	yes
rs1572666	0.002071701	699	0.102862106	0.738057778	179628	0.021626016	yes
rs3858032	0.002072581	700	-0.102866242	0.557303395	133231	-0.04097561	yes
rs10861910	0.002073368	701	0.096876926	0.222876854	50433	-0.079512195	no
rs11074795	0.002074325	702	-0.097890819	0.811878017	198592	0.016491228	no
rs7221510	0.002075146	703	0.084230532	0.532360359	127034	-0.029828609	no
rs2873938	0.002081283	704	0.074635299	0.051533975	10305	0.051252472	yes
rs7961807	0.002090615	705	-0.091935484	0.762806574	185959	0.021025641	no
rs4597724	0.00209389	706	-0.109231533	0.392933153	92003	-0.035266974	yes
rs13206666	0.002096947	707	-0.101725909	0.573330415	137205	0.03495935	no
rs4936253	0.002102734	708	0.106039369	0.978759653	241950	-0.001812788	no
rs1077580	0.002103005	709	-0.109739256	0.072964671	15126	0.118918919	no
rs4977761	0.002108088	710	0.102229299	0.466266268	110214	-0.049166667	no
rs4447803	0.002108556	711	-0.095931785	0.929671544	229029	-0.005833333	yes
rs336543	0.002109621	712	-0.108413807	0.447316484	105421	-0.042113821	yes
rs2153324	0.00210993	713	0.097246764	0.412817356	96926	-0.052687039	no
rs250377	0.002110686	714	0.042821808	0.739710446	179943	-0.023458904	no
rs10826574	0.002114263	715	-0.107868968	0.95350172	235279	-0.004009355	yes
rs2259573	0.002115887	716	0.103462092	0.06316993	12875	-0.138536585	no
rs10951192	0.002117313	717	0.096658119	0.633314009	152848	-0.031641397	no
rs3849524	0.002118841	718	-0.109822167	0.283375271	64908	-0.066413975	yes
rs2269781	0.002131015	719	-0.102496404	0.691419553	167471	-0.027642276	yes
rs12202881	0.00214213	720	-0.1053141	0.658393166	159293	-0.028455285	yes
rs10250550	0.002144228	721	-0.104417506	0.638951507	154307	-0.032404181	yes
rs7914830	0.002151053	722	-0.100889557	0.026133319	5048	0.149828767	no
rs1076116	0.002155208	723	0.104482614	0.429468242	101040	0.057498198	yes
rs4546489	0.002156632	724	-0.101684816	0.573330415	137185	0.03495935	no
rs9370435	0.002156632	725	-0.101684816	0.536084396	127885	0.038562953	no
rs10948931	0.002156632	726	-0.101684816	0.573330415	137194	0.03495935	no
rs36735	0.002159429	727	-0.094427277	0.297681281	68257	-0.06601626	yes
rs17685702	0.00216079	728	0.094709266	0.225518135	51109	-0.076666667	no
rs353584	0.002162339	729	0.10480789	0.226777053	51522	0.082234674	yes
rs6684007	0.002165181	730	-0.101438258	0.163613996	36206	-0.097398374	yes
rs1393174	0.002169133	731	-0.041235834	0.468695531	110948	0.046666667	no
rs2804916	0.002172108	732	0.100267105	0.400478273	93850	0.0525	yes
rs4952479	0.002172979	733	-0.105146908	0.486294815	115254	-0.048780488	yes
rs2224184	0.002174051	734	0.004119581	0.28337508	64906	0.047179487	yes
rs1943918	0.002177197	735	-0.106707835	0.449668746	106087	0.051666667	no
rs12662480	0.002177747	736	-0.104376413	0.480927543	113978	-0.046341463	yes
rs2764862	0.002189673	737	0.107458393	0.608054531	146235	0.034634146	yes
rs9306510	0.002194472	738	-0.10584549	0.950310658	234346	-0.004065041	yes
rs17841064	0.00220243	739	0.096712554	0.100895716	21671	0.105	yes
rs7138971	0.002202868	740	0.100219298	0.015243292	2719	0.151120633	yes
rs13202490	0.002202908	741	-0.105155067	0.477786517	113178	-0.048617886	yes
rs1817608	0.002207491	742	-0.107350317	0.987358034	243967	0.001138211	no
rs13269700	0.002209203	743	-0.098353875	0.460408897	108948	0.053792182	no
rs1691255	0.002210212	744	-0.040647223	0.206390823	46547	-0.01356922	yes
rs339114	0.002211127	745	-0.106492706	0.525237511	125208	-0.047642276	yes
rs3112932	0.002212444	746	-0.103965482	0.639591473	154431	-0.03203252	yes
rs987532	0.002223004	747	0.096835833	0.61359123	147607	-0.030569106	no
rs6479906	0.002226266	748	0.106164669	0.381878101	89251	0.05784443	yes
rs858059	0.002240093	749	-0.104294226	0.669459873	162019	0.029166667	no
rs2800219	0.002240846	750	-0.034687357	0.204150629	45988	-0.015447154	yes
rs265784	0.002245502	751	0.047645058	0.946180234	233123	-0.004227642	no
rs2326478	0.002246925	752	0.097835721	0.616749484	148519	-0.034166667	no
rs2981167	0.002249552	753	-0.103513458	0.716309002	173971	0.023333333	no
rs1779969	0.002252764	754	-0.034343538	0.204150629	45993	-0.015447154	yes
rs6714884	0.002256402	755	-0.099564992	0.483637457	114590	-0.044444444	yes
rs1836648	0.002260489	756	-0.103595644	0.71675543	174054	-0.024166667	yes
rs1518845	0.002263341	757	-0.100834793	0.253267868	57693	0.079597891	no

rs168989	0.00226453	758	0.026534169	0.004330488	649	-0.135833333	no
rs7178378	0.002265287	759	-0.09685638	0.218076016	49412	-0.085365854	yes
rs9861194	0.00226736	760	0.102393672	0.322818383	74490	-0.060650407	no
rs10744636	0.002270497	761	0.103010068	0.359936673	83768	-0.062926829	no
rs7996197	0.002271692	762	-0.102463156	0.441840873	104091	-0.05374078	yes
rs11699936	0.002272057	763	-0.105393466	0.017885727	3248	-0.17398374	yes
rs2281791	0.002273428	764	0.098685022	0.169561469	37640	0.101666667	yes
rs10472952	0.002278017	765	-0.098941853	0.41059226	96365	-0.05203252	yes
rs2053526	0.002282973	766	0.102212572	0.933257775	229795	0.005136986	yes
rs2164029	0.002286915	767	0.10340101	0.56001022	133853	-0.036260163	no
rs10916171	0.002288483	768	-0.102373125	0.208589466	47084	-0.08195122	yes
rs16880836	0.002292495	769	-0.102578591	0.270868007	61986	0.022276423	no
rs10992658	0.002308958	770	-0.090404767	0.131379364	28511	0.004552846	no
rs2052028	0.002311404	771	-0.098787754	0.491875392	116689	0.049105691	no
rs1880762	0.002315202	772	0.102229177	0.06741992	13875	-0.111666667	no
rs1434588	0.002315656	773	-0.050765528	0.344363899	79841	0.066666667	no
rs1263951	0.002319779	774	0.09820674	0.200811833	45225	0.085691057	yes
rs10778671	0.002322474	775	0.077820012	0.660997662	159804	-0.03203252	no
rs893745	0.002324938	776	-0.016704335	0.367112635	85531	-0.016585366	yes
rs12148530	0.002328201	777	-0.103924389	0.704011366	170861	0.025379038	no
rs303958	0.002328683	778	0.101925231	0.828790826	202966	0.014189189	yes
rs9650048	0.00234121	779	0.091771112	0.920492483	226439	0.00601626	yes
rs6747497	0.002341588	780	0.096959112	0.691887539	167601	0.023739837	yes
rs9357402	0.002341758	781	-0.105566459	0.581439505	139441	0.038109756	no
rs4907999	0.00234659	782	0.006079404	0.652159644	157710	0.032300593	yes
rs2074121	0.002355679	783	0.002486131	0.309148381	71120	0.047804878	yes
rs6724520	0.002358964	784	-0.077583727	0.563483466	134738	-0.011056911	yes
rs2737214	0.002366239	785	-0.105434559	0.180440591	40236	-0.089756098	yes
rs6768562	0.002366904	786	0.106664169	0.791280273	193316	0.01804878	yes
rs10937074	0.002367695	787	0.106978908	0.296956664	68088	-0.069594595	no
rs10872606	0.002368066	788	-0.008701459	0.153399075	33736	-0.091666667	yes
rs10848028	0.002370591	789	-0.098654202	0.112484645	24145	-0.033170732	yes
rs17457220	0.002382314	790	-0.098294677	0.488692944	115873	0.050684932	no
rs5769986	0.002382326	791	0.093486747	0.304976203	69953	0.06	yes
rs6895297	0.002382766	792	0.100162358	0.283683034	64965	0.074159525	yes
rs140565	0.002383352	793	0.097742966	0.973480505	240427	0.002142386	yes
rs4800123	0.002383957	794	0.103802948	0.217915656	49371	-0.083445946	no
rs7639220	0.002384071	795	0.048804698	0.343591031	79691	-0.063356164	no
rs11208284	0.002385013	796	0.098346004	0.847355111	207997	-0.012845528	no
rs4952362	0.002389827	797	-0.098692974	0.675261274	163529	0.025853659	no
rs4419020	0.002394379	798	0.09822423	0.696886022	168863	-0.025337838	no
rs4712037	0.00239536	799	0.098592562	0.343910226	79769	0.059166667	yes
rs1487406	0.002398133	800	-0.101875709	0.826922156	202460	-0.015202703	yes
rs1461038	0.002400233	801	0.032987467	0.47727764	112982	0.046666667	yes
rs1553545	0.00240398	802	0.02196228	0.812480352	198796	-0.016422764	no
rs7586603	0.002405039	803	-0.098048079	0.787479167	192331	0.017303889	no
rs1981382	0.002412973	804	-0.104153355	0.288147233	66075	-0.073337788	yes
rs5750735	0.002424468	805	0.077861105	0.025639193	4906	0.032357724	yes
rs9382497	0.002433157	806	-0.100092459	0.573330415	137211	0.03495935	no
rs1551410	0.002434607	807	-0.102249846	0.483931756	114674	-0.047972973	yes
rs1263965	0.002437066	808	0.099809137	0.014426087	2525	0.064552846	yes
rs820505	0.002444111	809	0.09528457	0.236132565	53709	0.080916282	yes
rs13250256	0.002449368	810	-0.102907335	0.489808681	116261	-0.045203252	yes
rs12692909	0.002450579	811	-0.101962194	0.465386941	110022	0.028345419	no
rs2371183	0.002453798	812	0.101216119	0.236661004	53805	-0.072845528	no
rs6825174	0.002460158	813	0.066632422	0.100549227	21554	-0.114864865	no
rs9921222	0.002463981	814	0.081345975	0.665726004	161043	0.031947784	yes
rs6743542	0.002467343	815	0.095244003	0.88323999	217145	0.009081197	yes
rs560162	0.002470139	816	0.013048325	0.84943156	208426	-0.013354701	no
rs11596463	0.002475291	817	-0.098705568	0.641344932	154810	0.030406504	no
rs10841417	0.002476582	818	-0.056759811	0.066445512	13601	-0.070894309	yes
rs6544416	0.002479527	819	-0.098089172	0.675261274	163538	0.025853659	no
rs6544417	0.002479527	820	-0.098089172	0.675261274	163540	0.025853659	no
rs2737205	0.002489279	821	-0.105681118	0.116136067	25031	-0.107479675	yes
rs12433772	0.002496873	822	-0.095438669	0.719678518	174822	-0.024065041	yes
rs17774601	0.002501868	823	0.100600886	0.702277221	170215	0.022280702	yes
rs1154862	0.002507009	824	0.097959394	0.72898777	177279	-0.024065041	no
rs991389	0.002512571	825	-0.063170331	0.070476232	14482	0.03	no
rs10440835	0.002514491	826	0.097370043	0.200576192	45143	-0.089593496	no

rs6924349	0.002515702	827	0.0425827	0.563670957	134798	-0.038068556	no
rs11593406	0.002516309	828	0.079453462	0.442580646	104333	0.048943089	yes
rs10740696	0.002516309	829	0.079453462	0.442580646	104339	0.048943089	yes
rs11624647	0.002518626	830	-0.101393829	0.182858744	40839	0.095608108	no
rs2756132	0.002521495	831	0.095686161	0.07568121	15697	0.113052076	yes
rs2985867	0.00252179	832	0.105866037	0.608054531	146246	0.034634146	yes
rs11741701	0.002523785	833	0.095983152	0.420743915	98863	0.052520325	yes
rs7470287	0.002529302	834	-0.066299083	0.181004699	40376	-0.090894309	yes
rs7502120	0.002533761	835	-0.104170947	0.543457867	129779	0.040064103	no
rs2835944	0.002536479	836	-0.102537497	0.660980699	159756	0.031219512	no
rs6538524	0.002538593	837	0.102599137	0.183023974	40883	-0.087317073	no
rs11998040	0.002544085	838	0.102298124	0.545612789	130357	-0.040813008	no
rs969930	0.002545874	839	0.101865402	0.831270421	203635	-0.014146341	no
rs2929563	0.002546317	840	-0.101643723	0.44064719	103751	-0.049593496	yes
rs2923408	0.002547576	841	-0.051982741	0.623795791	150344	-0.035447154	yes
rs9296791	0.00255022	842	-0.100092459	0.573330415	137182	0.03495935	no
rs4275061	0.00255022	843	-0.100092459	0.573330415	137187	0.03495935	no
rs9475328	0.00255022	844	-0.100092459	0.785698415	191899	0.016666667	no
rs7751266	0.00255022	845	-0.100092459	0.641679838	154948	0.029166667	no
rs10948927	0.00255022	846	-0.100092459	0.573330415	137219	0.03495935	no
rs1453380	0.00255882	847	0.016940621	0.168188562	37319	0.074471545	yes
rs7979286	0.002560056	848	0.084271625	0.102796612	21990	-0.11300813	no
rs10069050	0.002560117	849	0.028169303	0.812480352	198798	-0.016422764	no
rs1360877	0.002561548	850	0.099553313	0.967658754	238892	0.002601626	yes
rs13056578	0.002561816	851	-0.095644134	0.666841526	161249	0.028333333	no
rs12491503	0.002562332	852	0.098839121	0.235797929	53638	0.080162602	yes
rs7972505	0.002562885	853	0.101212246	0.534603062	127517	0.041549296	yes
rs2794233	0.002563819	854	-0.101962194	0.063913459	13029	0.056747967	no
rs1586070	0.002567228	855	-0.102126567	0.758742633	184866	0.020162602	no
rs10836637	0.002569935	856	0.103616191	0.090025285	19098	-0.108292683	no
rs9291789	0.002571868	857	0.099126772	0.605246245	145365	-0.036422764	no
rs1269480	0.002571883	858	-0.037528251	0.738366236	179709	-0.022113821	yes
rs4522182	0.002572222	859	-0.097318677	0.592303972	142207	0.035343035	no
rs7817958	0.002573134	860	0.093075817	0.691500891	167520	0.026388889	yes
rs10444992	0.002574581	861	0.091565646	0.95759397	236212	-0.003333333	no
rs2078305	0.00258056	862	-0.095397576	0.588357296	141286	0.036910569	no
rs10828975	0.002583051	863	-0.096306306	0.501233735	119140	-0.046473303	yes
rs10906224	0.002591479	864	-0.098582289	0.641344932	154762	0.030406504	no
rs2056642	0.002593018	865	-0.104946924	0.107941989	23213	-0.10890411	yes
rs4800784	0.002605021	866	-0.10714742	0.987358034	243946	0.001138211	no
rs1354723	0.002606382	867	0.097557456	0.78577569	191916	-0.018333333	no
rs9991261	0.002608734	868	-0.103453267	0.594480148	142761	0.034634146	no
rs1570542	0.002612371	869	-0.100927548	0.327635597	75738	-0.070412518	yes
rs2352776	0.002613899	870	-0.099476063	0.83171186	203775	0.014634146	no
rs6543606	0.002615348	871	0.098676592	0.488763243	115901	0.042847726	yes
rs1625785	0.002619261	872	-0.101422241	0.942876526	232538	-0.004807692	yes
rs2119261	0.002627033	873	-0.097825415	0.457635477	108012	0.050675676	no
rs9917659	0.002628345	874	0.098346004	0.819598212	200482	0.013821138	yes
rs413420	0.002628665	875	0.03139511	0.72613871	176499	0.02505847	yes
rs909073	0.002632567	876	0.062030195	0.812418316	198759	0.015853659	yes
rs303953	0.0026346	877	0.099735648	0.635741117	153480	0.030652604	yes
rs1358855	0.002639057	878	0.050626669	0.95024632	234315	0.004166667	yes
rs4961077	0.002639765	879	-0.102284946	0.149965253	32938	0.094471545	no
rs4247680	0.002641159	880	-0.096258889	0.712037492	172768	0.023577236	no
rs2108600	0.002642216	881	0.061937264	0.801215652	195962	0.016747967	yes
rs9572788	0.002657057	882	0.100843837	0.872545476	214389	0.010406504	yes
rs2117545	0.002659012	883	-0.103583916	0.39996004	93692	-0.057363014	yes
rs12205102	0.00267259	884	0.084271625	0.225040136	51013	0.087837838	yes
rs17271123	0.00267717	885	-0.103842203	0.193115875	43398	0.0875	no
rs6807691	0.002680211	886	-0.101962194	0.741880343	180573	0.021588662	no
rs2925712	0.002685834	887	0.106687898	0.721754899	175396	-0.024166667	no
rs1160350	0.002688596	888	-0.10163345	0.544150359	129889	-0.042113821	yes
rs321476	0.002695536	889	-0.096267577	0.676157417	163791	0.028333333	no
rs7487222	0.002695914	890	0.09201767	0.6440445	155621	-0.028333333	no
rs2460177	0.002699114	891	-0.100971878	0.86604482	212683	-0.011206328	yes
rs1865998	0.002699145	892	0.093430891	0.185726784	41576	0.085273973	yes
rs7540934	0.002699787	893	-0.105188001	0.340354642	78901	-0.066341463	yes
rs1986745	0.002700901	894	-0.101818789	0.734492545	178686	0.022051282	no
rs3799925	0.002702421	895	-0.095207668	0.049556505	9859	0.082276423	no

rs426776	0.002703936	896	0.099835103	0.896414539	220423	0.008130081	yes
rs9402292	0.002709035	897	0.100760222	0.518951846	123605	0.045691057	yes
rs9600563	0.002710147	898	0.104290278	0.38218956	89316	-0.0625	no
rs4573896	0.002713115	899	0.097894409	0.785518278	191854	-0.017039759	no
rs7768633	0.002719147	900	-0.099245244	0.536084396	127886	0.038562953	no
rs11720491	0.002725192	901	-0.105640025	0.935556992	230408	-0.005603164	yes
rs753426	0.002734143	902	0.100608059	0.79532099	194367	-0.017723577	no
rs11099878	0.002735132	903	-0.104006575	0.337870444	78258	-0.066743573	yes
rs2661249	0.002735342	904	0.06693806	0.604218734	145157	-0.037310743	no
rs4959631	0.002741923	905	0.095500308	0.766868573	186995	-0.018699187	no
rs1077581	0.002742285	906	-0.106533799	0.206259923	46523	0.083076923	no
rs2072633	0.002744063	907	-0.100945141	0.553473702	132205	0.04109589	no
rs1561030	0.002746473	908	0.099743168	0.925186558	227955	-0.006341463	no
rs611708	0.002750675	909	-0.091997123	0.600370397	144273	-0.034308943	yes
rs2283433	0.002755956	910	0.028598015	0.842039604	206571	0.013513514	yes
rs339676	0.00275977	911	-0.108208342	0.377336111	88183	0.064552846	no
rs2295283	0.002763771	912	-0.098623382	0.136619465	29760	0.098220171	no
rs6558646	0.002776362	913	-0.023073762	0.386490078	90491	0.062276423	no
rs12235034	0.002777847	914	0.096270803	0.886564509	217970	-0.009756098	no
rs4793550	0.002789389	915	0.09692928	0.399321358	93505	0.054796748	yes
rs7751000	0.002791817	916	-0.102907335	0.374301258	87319	0.056910569	no
rs6578233	0.002796586	917	0.096753647	0.35406059	82314	0.059166667	yes
rs9838703	0.002798554	918	-0.099763715	0.077154382	16069	-0.11398374	yes
rs2152694	0.002818713	919	0.103297719	0.457510226	107988	0.048287671	yes
rs7942520	0.002826284	920	0.00093985	0.212470908	48016	0.043177324	yes
rs501089	0.002829516	921	0.101960784	1	246924	0	no
rs504560	0.002830361	922	0.092765124	0.591366338	141944	-0.034580688	no
rs2725627	0.002830645	923	0.099619889	0.155135335	34209	0.094634146	yes
rs7708643	0.002831914	924	0.010530101	0.616091082	148382	-0.036585366	no
rs17785113	0.002833021	925	-0.098294637	0.098126703	20914	0.102601626	no
rs7222497	0.002836487	926	0.102249874	0.842621213	206707	0.014471545	yes
rs1337719	0.002848856	927	0.10275905	0.06927435	14259	0.121232877	yes
rs2862294	0.002858997	928	-0.092541607	0.634749835	153197	0.030406504	no
rs7341502	0.002881414	929	0.081479387	0.100308618	21490	0.068466899	yes
rs10142564	0.002891167	930	0.004119581	0.164027127	36285	-0.027642276	no
rs1732318	0.002902736	931	-0.100297925	0.759996529	185195	0.019932432	no
rs1395347	0.002902736	932	-0.100297925	0.759996529	185196	0.019932432	no
rs1887139	0.002910612	933	0.098526474	0.035040879	6869	0.13804878	yes
rs10869409	0.00291267	934	-0.095156137	0.449680523	106099	-0.048648649	yes
rs7452170	0.002927241	935	-0.098541196	0.502392504	119440	0.041626016	no
rs8041943	0.002927296	936	0.101828642	0.70899774	172096	-0.025203252	no
rs2616973	0.002927735	937	0.025509233	0.523458873	124823	-0.047462096	no
rs3847418	0.002933271	938	-0.024707212	0.708839166	172005	0.001138211	no
rs1909333	0.002940538	939	-0.099845901	0.420054943	98689	0.049796748	no
rs2005775	0.002948614	940	0.094466044	0.857883334	210726	0.012520325	yes
rs688480	0.002950351	941	-0.099670205	0.887680551	218211	0.009268293	no
rs914394	0.002950671	942	-0.101674543	0.069210426	14252	-0.122972973	yes
rs7001238	0.002955556	943	0.100320513	0.458872903	108435	0.044715447	yes
rs5002199	0.002967562	944	-0.093846312	0.719678518	174826	-0.024065041	yes
rs1951601	0.002967562	945	-0.093846312	0.719678518	174829	-0.024065041	yes
rs17257138	0.002967562	946	-0.093846312	0.719678518	174847	-0.024065041	yes
rs10255204	0.002981406	947	-0.096743374	0.90186302	221784	-0.008547009	yes
rs16880781	0.002981685	948	-0.091072529	0.64759594	156473	0.030406504	no
rs84853	0.002982726	949	0.008623542	0.40184733	94207	-0.06097561	no
rs3825415	0.002983113	950	-0.048756934	0.49669055	117868	0.047222222	no
rs7665044	0.002984849	951	-0.101109513	0.330026073	76354	0.063447594	no
rs2851162	0.002987882	952	0.09528457	0.712844847	172976	-0.02504065	no
rs2256383	0.002992681	953	0.058670639	0.769639793	187649	-0.006756757	no
rs7798509	0.002998665	954	-0.099311691	0.747170467	181851	-0.0225	yes
rs9466956	0.003001817	955	0.095490035	0.490005566	116325	0.04699187	yes
rs12712786	0.003007087	956	-0.101797822	0.292707073	67234	-0.074146341	yes
rs17629265	0.003007087	957	-0.101797822	0.18836897	42176	-0.078699187	yes
rs7697556	0.003016113	958	0.058629546	0.25728662	58629	0.02966381	yes
rs17836920	0.003016794	959	0.095572221	0.630561368	152053	-0.035689536	no
rs10825915	0.003020893	960	0.097452229	0.896333022	220406	-0.008108108	no
rs10430146	0.003021914	961	0.094961076	0.846766255	207838	-0.012820513	no
rs9469914	0.003026636	962	0.096640214	0.286141867	65582	-0.072181938	no
rs4724999	0.003030374	963	0.09786316	0.903986451	222284	0.00796748	yes
rs932768	0.003030888	964	0.098150812	0.88619064	217837	-0.009417808	no

rs10246303	0.003032991	965	0.10675049	0.249938858	56828	0.076403326	yes
rs12487188	0.003035836	966	0.093363468	0.64759594	156487	0.030406504	yes
rs2111546	0.003037891	967	0.097575509	0.463437146	109579	0.044166667	yes
rs2617100	0.003040483	968	0.100170663	0.127906128	27738	-0.109166667	no
rs2794236	0.003040501	969	-0.10053421	0.041282258	8150	0.044552846	no
rs10951406	0.003041277	970	0.089521266	0.031002444	6039	0.132682927	yes
rs1563786	0.003043899	971	-0.103318561	0.881562338	216719	-0.010894309	yes
rs7168186	0.003047428	972	0.098102263	0.68222114	165235	-0.028512539	no
rs7591919	0.003048264	973	0.000975961	0.916979402	225583	-0.007642276	no
rs10756632	0.003050674	974	0.101109843	0.315881172	72781	-0.067479675	no
rs2398829	0.003054458	975	-0.087766068	0.248691518	56553	0.030555556	no
rs4964829	0.003057612	976	0.013755907	0.244294643	55547	0.07902439	yes
rs10097784	0.003064586	977	-0.102455311	0.14592305	31964	-0.105650685	yes
rs8047745	0.00306614	978	-0.014964444	0.332328255	76919	-0.0625	yes
rs7687844	0.003066403	979	0.104355866	0.07578213	15723	0.128378378	yes
rs12886205	0.003066864	980	-0.105566763	0.897018229	220537	0.009228741	no
rs10179167	0.003072556	981	-0.08240189	0.113535302	24428	0.057191781	no
rs16934224	0.003072611	982	-0.098827922	0.256994809	58521	0.070243902	no
rs7170719	0.00307988	983	-0.022580645	0.480907712	113955	-0.05	yes
rs12815313	0.003083323	984	-0.101290429	0.91417622	224927	-0.007363014	yes
rs3828324	0.003083476	985	-0.092038217	0.183941063	41096	-0.002113821	yes
rs12987376	0.003086001	986	0.101746456	0.991432185	245216	-0.000675676	no
rs10817700	0.003086622	987	-0.099484695	0.337087719	78046	0.061148649	no
rs1880763	0.003089932	988	0.099250051	0.078605464	16371	-0.106178862	no
rs2387659	0.003090458	989	-0.058280944	0.191350832	42951	-0.061486486	yes
rs2144749	0.003092736	990	-0.104006575	0.772713117	188472	-0.018127884	yes
rs7009280	0.00309542	991	-0.100525611	0.906426946	222890	-0.007642276	yes
rs1817607	0.003098827	992	-0.103288041	0.987358034	243966	0.001138211	no
rs213018	0.003100771	993	-0.048756934	0.503333477	119629	-0.022886736	yes
rs1364002	0.00310625	994	0.090889416	0.964483206	238086	0.002764228	yes
rs11147298	0.003107149	995	0.09418533	0.897001537	220529	0.009268293	yes
rs5754086	0.003108638	996	0.10166427	0.371128897	86644	-0.009918699	no
rs6071775	0.003116377	997	0.100154099	0.100069005	21411	-0.107119314	no
rs3825991	0.003119627	998	0.069858229	0.732424196	178105	-0.023071852	no
rs2230808	0.00312166	999	0.092315328	0.7137641	173290	-0.022764228	no
rs12876129	0.003124066	1000	0.10154099	0.675979357	163712	-0.028378378	no

Table S3(b).The 30 SNPs that also have a raw P-value <0.05 for the validation dataset.

SNP.ID	raw p-value in GSE6	rank in GSE65777	MAF difference in (	raw p-value in GSE6	rank in GSE66903	MAF difference in (	of MAF difference?
rs2839629	7.81E-06	4	0.093538114	0.01734138	3164	0.110135135	yes
rs41485553	0.000618304	216	-0.118625437	0.020865262	3890	-0.146901709	yes
rs8134673	0.000643405	222	-0.116416684	0.014339415	2515	-0.093050451	yes
rs10818963	0.000667007	231	0.117012533	0.021928223	4139	-0.158373984	no
rs8127441	0.000778462	264	-0.114824327	0.006019724	938	-0.120246479	yes
rs7504457	0.001071351	369	0.104797617	0.036403739	7136	-0.136824324	no
rs13044355	0.001105626	383	-0.111434148	0.039246554	7726	-0.154166667	yes
rs4720121	0.001282137	442	-0.102054654	0.024525445	4669	0.14796748	no
rs322838	0.001359797	470	0.101401628	0.012819203	2213	0.166829268	yes
rs12700629	0.001363149	472	-0.022875817	0.027495327	5304	-0.139492148	yes
rs6575988	0.001564041	531	0.101653996	0.043483328	8603	0.132290185	yes
rs2213736	0.001589397	538	0.111382782	0.012533265	2157	-0.179512195	no
rs6973092	0.001746022	591	0.099031024	0.036373969	7127	0.150081301	yes
rs1536651	0.001761785	597	-0.104859256	0.016579648	2983	-0.11300813	yes
rs322826	0.001861728	621	0.102392784	0.019012573	3489	0.159459459	yes
rs6075977	0.001868018	625	0.10590713	0.040329842	7933	0.142276423	yes
rs8036757	0.001891985	632	-0.028906984	0.028213343	5479	-0.063089431	yes
rs2251671	0.001966844	657	-0.103578467	0.008980469	1487	-0.180189673	yes
rs7776738	0.002001706	673	-0.043938771	0.018115614	3293	-0.146341463	yes
rs10818964	0.002001793	674	0.105198941	0.043575611	8623	-0.137112722	no
rs7914830	0.002151053	722	-0.100889557	0.026133319	5048	0.149828767	no
rs7138971	0.002202868	740	0.100219298	0.015243292	2719	0.151120633	yes
rs168989	0.00226453	758	0.026534169	0.004330488	649	-0.135833333	no
rs11699936	0.002272057	763	-0.105393466	0.017885727	3248	-0.17398374	yes
rs5750735	0.002424468	805	0.077861105	0.025639193	4906	0.032357724	yes
rs1263965	0.002437066	808	0.099809137	0.014426087	2525	0.064552846	yes
rs3799925	0.002702421	895	-0.095207668	0.049556505	9859	0.082276423	no
rs1887139	0.002910612	933	0.098526474	0.035040879	6869	0.13804878	yes
rs2794236	0.003040501	969	-0.10053421	0.041282258	8150	0.044552846	no
rs10951406	0.003041277	970	0.089521266	0.031002444	6039	0.132682927	yes
